# McvR, a single domain response regulator regulates motility and virulence in the plant pathogen *Xanthomonas campestris*

**DOI:** 10.1111/mpp.13186

**Published:** 2022-02-13

**Authors:** Rui‐Fang Li, Pei‐Dong Ren, Qian‐Qian Liu, Jia‐Li Yao, Liu Wu, Gui‐Ning Zhu, Xiao‐Yong Xian, Ji‐Liang Tang, Guang‐Tao Lu

**Affiliations:** ^1^ Guangxi Key Laboratory of Biology for Crop Diseases and Insect Pests Plant Protection Research Institute Guangxi Academy of Agricultural Sciences Nanning China; ^2^ State Key Laboratory for Conservation and Utilization of Subtropical Agro‐bioresources College of Life Science and Technology Guangxi University Nanning China

**Keywords:** co‐operate, motility, regulatory, single‐domain response regulator, *Xanthomonas*

## Abstract

Signal transduction pathways mediated by sensor histidine kinases and cognate response regulators control a variety of physiological processes in response to environmental conditions in most bacteria. Comparatively little is known about the mechanism(s) by which single‐domain response regulators (SD‐RRs), which lack a dedicated output domain but harbour a phosphoryl receiver domain, exert their various regulatory effects in bacteria. Here we have examined the role of the SD‐RR proteins encoded by the phytopathogen *Xanthomonas campestris* pv. *campestris* (Xcc). We describe the identification and characterization of a SD‐RR protein named McvR (motility, chemotaxis, and virulence‐related response regulator) that is required for virulence and motility regulation in Xcc. Deletion of the *mcvR* open reading frame caused reduced motility, chemotactic movement, and virulence in Xcc. Global transcriptome analyses revealed the McvR had a broad regulatory role and that most motility and pathogenicity genes were down‐regulated in the *mcvR* mutant. Bacterial two‐hybrid and protein pull‐down assays revealed that McvR did not physically interact with components of the bacterial flagellum but interacts with other SD‐RR proteins (like CheY) and the subset of DNA‐binding proteins involved in gene regulation. Site‐directed mutagenesis and phosphor‐transfer experiments revealed that the aspartyl residue at position 55 of the receiver domain is important for phosphorylation and the regulatory activity of McvR protein. Taken together, the findings describe a previously unrecognized class of SD‐RR protein that contributes to the regulation of motility and virulence in Xcc.

## Graphical abstract

McvR modulates cell motility via interacting with CheY protein and indirectly regulating the expression of a series of motility‐related genes.

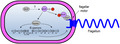

## 1 INTRODUCTION

Bacteria constantly monitor their environment to ensure survival and successful proliferation. The adaptive responses often involve motility, the formation of biofilms, or the activation of mechanisms that negate stress. Two‐component signalling systems (TCSs) are common mechanisms that mediate bacterial adaption to surrounding changes. A prototypical TCS is composed of a membrane‐associated sensor histidine kinase, which is capable of sensing a specific stimulus and autophosphorylation on a conserved histidine residue in the transmitter domain, and a response regulator that interacts with the phosphorylated sensor kinase. During the interaction, the sensor kinase transfers the phosphoryl group to the conserved aspartate residue in the N‐terminal receiver domain of the response regulator, leading to a conformational change and the activation of its C‐terminal output domain (Buschiazzo & Trajtenberg, 2019; Gao et al., 2019; Stock et al., 2000).

Generally, response regulators (RRs) are composed of an N‐terminal (phosphoryl‐) receiver domain and a variable C‐terminal output domain that generates a wide variety of cellular responses to the corresponding environmental signals (Gao et al., 2019). However, some RRs, the so‐called single‐domain response regulators (SD‐RRs), harbour a receiver (REC) domain only. The number of SD‐RRs usually correlates with the genome size and the total number of TCS proteins. Certain bacteria, such as *Myxococcus xanthus*, harbour more than 40 SD‐RRs (Müller et al., 2013). In the Pfam database, approximately one‐quarter (23%) of RRs belong to the SD‐RR group (Gao et al., 2019). Although SD‐RRs are widespread in bacteria and form the second largest class of response regulators, knowledge about their cellular functions is poor. So far, the best characterized SD‐RR is the chemotaxis protein CheY from *Escherichia coli* (Parkinson et al., 2015; Sarkar et al., 2010). CheY and the majority of CheY homologs in other organisms have been shown to regulate cell swimming motility by protein–protein interactions depending on the RR’s phosphorylation state. When signals from transmembrane chemoreceptors are received, the sensor kinase CheA transfers a phosphoryl group to CheY, and phospho‐CheY (CheY‐P) interacts with the FliM/FliN component of the flagellar motor switch complex and causes a reversal in flagellar rotation from counter clockwise to clockwise. As a consequence, the cell stops swimming and the cell body rotates into a new, random orientation, which is called a “tumble”. Additionally, CheY homologs (CheY‐like) in certain bacteria (including *Cytophaga* and *Flavobacterium*) have been shown to control twitching and gliding motility. These motility systems are not homologous to bacterial flagella and neither of them harbours FliM‐related proteins (Jarrell & McBride, 2008). There are only a few reports about specific functions of SD‐RRs other than control of motility; examples include Spo0F in *Bacillus subtilis* and DivK in *Caulobacter crescentus*, which act as a shuttle or a sink, which mediates the phosphoryl group in a phosphorelay or drains phosphate away from histidine kinases (Paul et al., 2008; Tzeng et al., 1998).

The gram‐negative bacterium *Xanthomonas campestris* pv. *campestris* (Xcc) is the causal agent of black rot disease. This pathogen can cause disease in almost all members of the crucifer family (*Brassicaceae*), including important vegetables such as broccoli, Brussels sprouts, cabbage, cauliflower, kale, mustard and radish, oilseed rape, and the model plant *Arabidopsis thaliana* (Vicente & Holub, 2013). Xcc infects host plants via wounds or hydathodes. After infection, the bacterial cells multiply in the intercellular spaces, spreading via the vascular system, and leading to the development of disease symptoms: vein blackening and V‐shaped chlorotic and necrotic lesions extending from leaf margins along veins (Chan & Goodwin, 1999). The virulence of Xcc toward plants depends on a number of pathogenic factors, including extracellular enzymes (such as cellulase, protease, and amylase), extracellular polysaccharide (EPS) and lipopolysaccharides, type III effector proteins, and biofilm formation (Ryan et al., 2011).

Over recent decades, Xcc has been an important model for studying bacterial plant infection. The entire genome sequences of three Xcc strains have been determined (Qian et al., 2005; da Silva et al., 2002; Vorhölter et al., 2008), and each of the genomes encodes 32 histidine kinase sensors, 54 response regulators, and 20 histidine‐containing phosphotransfer domain (HPt) proteins. Among the 54 response regulators, 16 belong to the SD‐RR group. Several TCSs have been shown to regulate Xcc virulence and various phenotypes. The TCS RpfC/RpfG positively controls the activity of extracellular enzymes and the production of EPS, and negatively regulates the biosynthesis of a diffusible signal factor (*cis*‐11‐methyl‐2‐dodecenoic acid) that functions in cell‐to‐cell communication (Ryan et al., 2007; Tang et al., 1991). ColS_XC1050_/ColR_XC1049_ regulates several cellular processes, including cell proliferation, hypersensitive response, and stress tolerance (Zhang et al., 2008). RavS/RavR regulates EPS synthesis, biofilm production, and motility via the control of cellular concentrations of cyclic‐di‐guanosine monophosphate (c‐di‐GMP) and the activity of the global regulator Clp (He et al., 2009). HpaS/HrpG positively controls the expression of the type III secretion system (Li et al., 2014). Beside these TCSs, SD‐RRs VemR and CheY have also been characterized. VemR positively regulates virulence and adaptation, and controls swimming motility via interacting with the flagellum basal body protein FliM (Li et al., 2020; Tao & He, 2010). CheY, similar to its counterpart in *E. coli*, regulates motility via binding to the flagellar motor (Li et al., 2020). Here, we demonstrate that a SD‐RR named McvR (motility, chemotaxis and virulence related response regulator) is involved in cell motility, chemotaxis and virulence in Xcc. Moreover, our data also reveal that, unlike the CheY and the majority of CheY‐like proteins that regulate cell motility via binding to the flagellar motor switch complex, McvR might co‐operate with CheY and certain DNA‐binding proteins involved in controlling gene expression to regulate cell motility and chemotaxis.

## 2 RESULTS

### 2.1 McvR is involved in swimming motility, chemotaxis, and virulence

Scanning of the sequenced genomes of three Xcc strains (American Type Culture Collection [ATCC] 33913, 8004, and B100) revealed that 16 open reading frames (ORFs) in each of the strains were predicted to encode SD‐RRs. In our earlier work, we aimed to investigate the functions of these SD‐RRs in Xcc; therefore, we constructed a series of insertional mutants using a suicide vector (pK18*mob*) strategy (Schäfer et al., 1994). In previous work we screened these mutants for influence on cell motility and showed that several of them influenced motility, including disruptions to the known regulators CheY and VemR (Li et al., 2020; Qi et al., 2020). Interestingly, insertional mutant strain 1966nk, which derived from the disruption of the ORF *XC_1966* (hereafter named *mcvR*) in the genome of Xcc strain 8004 (accession number CP000050), had reduced swimming ability when compared with the wild‐type Xcc strain. The *mcvR* gene encodes a protein of 123 amino acids (AAY49029.1). Amino acid sequence pairwise alignments using Vector NTI showed that McvR shares 17.7% identity and 36.9% similarity with the characterized SD‐RR CheY, and 9.4% identity and 24.4% similarity with VemR in Xcc (Figure S1), implying different roles of McvR, CheY, and VemR on cell motility in Xcc.

To facilitate further studies on the function of McvR in Xcc, we constructed in‐frame marker‐free deletion of the *mcvR* gene in the wild‐type strain 8004 via allelic homologous recombination employing the suicide plasmid pK18*mobsacB* (Schäfer et al., 1994), and designated the strain Δ*mcvR*. Simultaneously, a complemented strain was constructed by introducing a recombinant plasmid that was derived from the *mcvR*‐coding sequence, cloned into the vector pLAFR3, into the Δ*mcvR* strain. The resulting complemented strain was named CΔ*mcvR* (Table S1).

**FIGURE 1:**
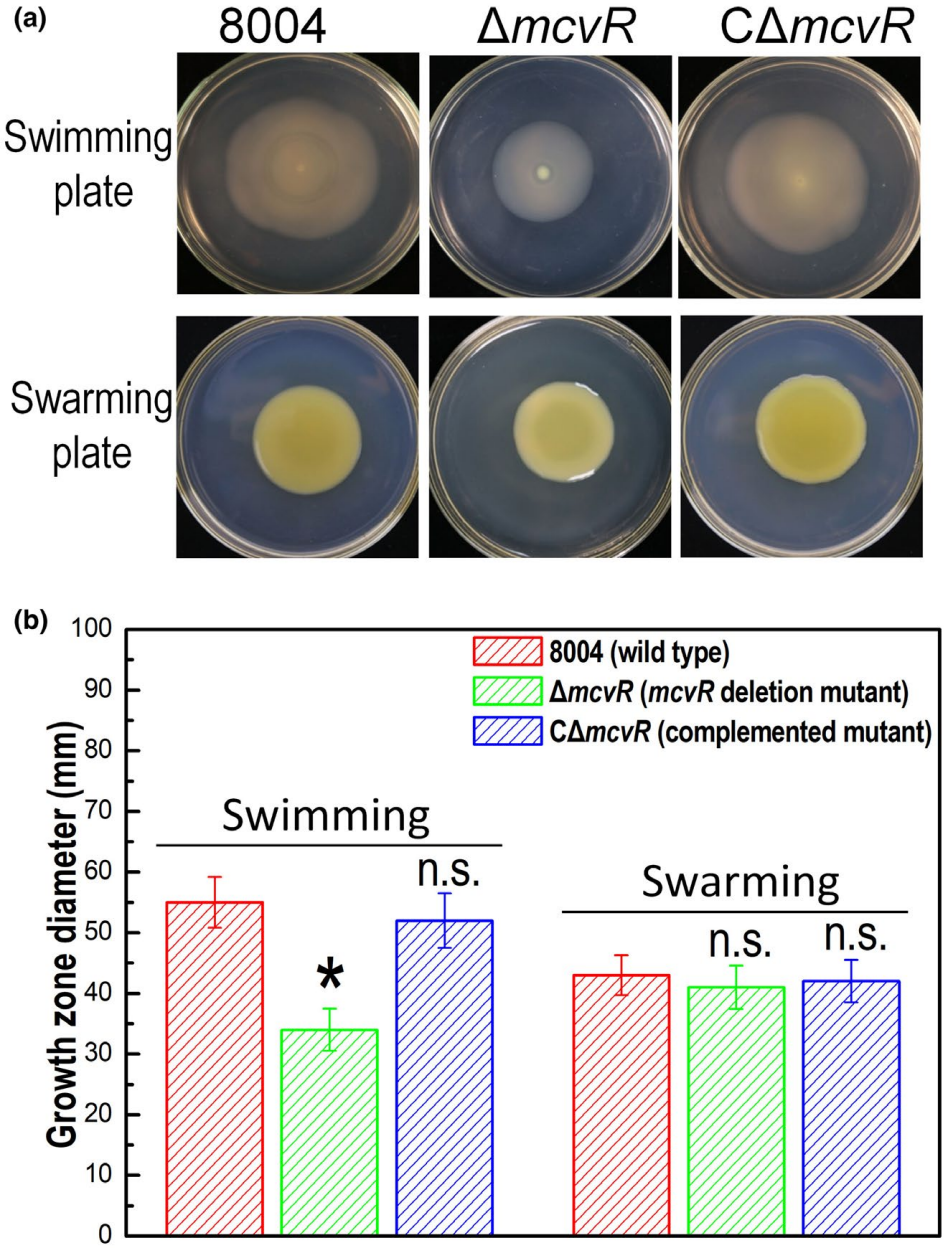
Effect of *mcvR* deletion on *Xanthomonas* *campestris* pv. *campestris* (Xcc)motility. Xcc strains were stabbed into “swim” plates or inoculated onto “swarm” plates followed by incubation for 4 or 3 days, respectively. The representative colony morphologies of Xcc strains were photographed (a) and the colony diameters of each strain on the different media were measured (b). Values given are the mean and *SD* (standard deviation) from 10 measurements in a representative experiment. Significance was determined by analysis of variance (ANOVA) and Dunnett's post hoc test for comparison to the wild type. *p < 0.05; n.s., not significant. Similar results were obtained in two other independent experiments

To confirm that deletion of *mcvR* influences cell motility in Xcc, the newly constructed strains were tested by inoculation on swimming plates (0.28% wt/vol agar) and swarming plates (0.6% wt/vol agar). The results showed that the Δ*mcvR* mutant displayed diminished swimming capacity (reduced by approximately 62%) compared to the wild type, although the mutant had similar swarming capacity as the wild type (Figure 1). The swimming motility of Δ*mcvR* was restored by complementation (Figure 1).

These findings prompted us to test the role of McvR in Xcc chemotaxis. To do this, we performed a syringe capillary assay to compare the chemotactic response of Xcc wild‐type strain and the Δ*mcvR* mutant to a variety of agents. The panel of chemotactic agents comprised four inorganic salts (NaCl, CaCl_2_, MgCl_2_, CuSO_4_), six carbohydrates (xylose, galactose, glucose, fructose, sucrose, maltose), three organic acids (citric acid, succinic acid, malic acid) and 10 amino acids (serine, threonine, methionine, phenylalanine, arginine, alanine, leucine, lysine, valine, glutamine).

**FIGURE 2:**
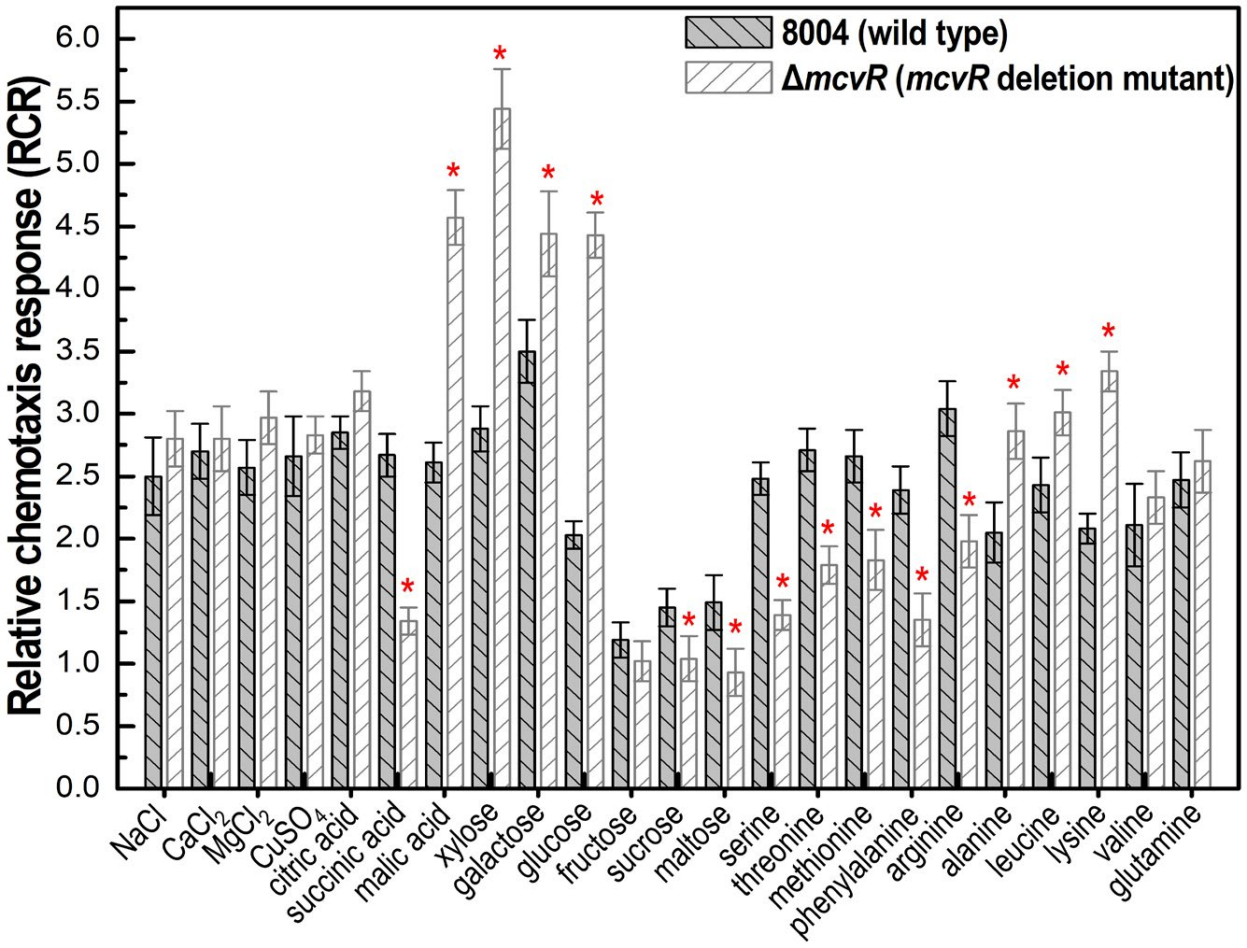
Quantitative chemotaxis assay for the Δ*mcvR* mutant with different chemotactic agents. The relative chemotaxis response (RCR) was determined as the ratio of the number of bacterial cells in the test capillary containing the chemotactic agent to the number of bacterial cells in the capillary containing phosphate‐buffered saline. Values given are the mean ± *SD* of five measurements from a representative experiment. The asterisk on each column indicates significantly different values compared with the value of the wild type (Student's *t* test; *p* < 0.05). Similar results were obtained in two other independent experiments

As shown in Figure 2, the Δ*mcvR* strain exhibited a significantly reduced chemotactic movement towards succinic acid, sucrose, maltose, serine, threonine, methionine, phenylalanine, and arginine, and significantly stronger chemotactic movement towards malic acid, xylose, galactose, glucose, alanine, leucine, and lysine compared with the wild type (*p* < 0.05, *t* test**)**, indicating that McvR regulates positively or negatively the chemotactic response to different agents in Xcc.

We examined other phenotypes such as the production of EPS and the extracellular enzymes cellulase, amylase, and protease, which collectively contribute to virulence in Xcc during the disease process. However, no differences were seen between Δ*mcvR* and the wild type, indicating that McvR had no influence on these phenotypes (Figure S2).

**FIGURE 3:**
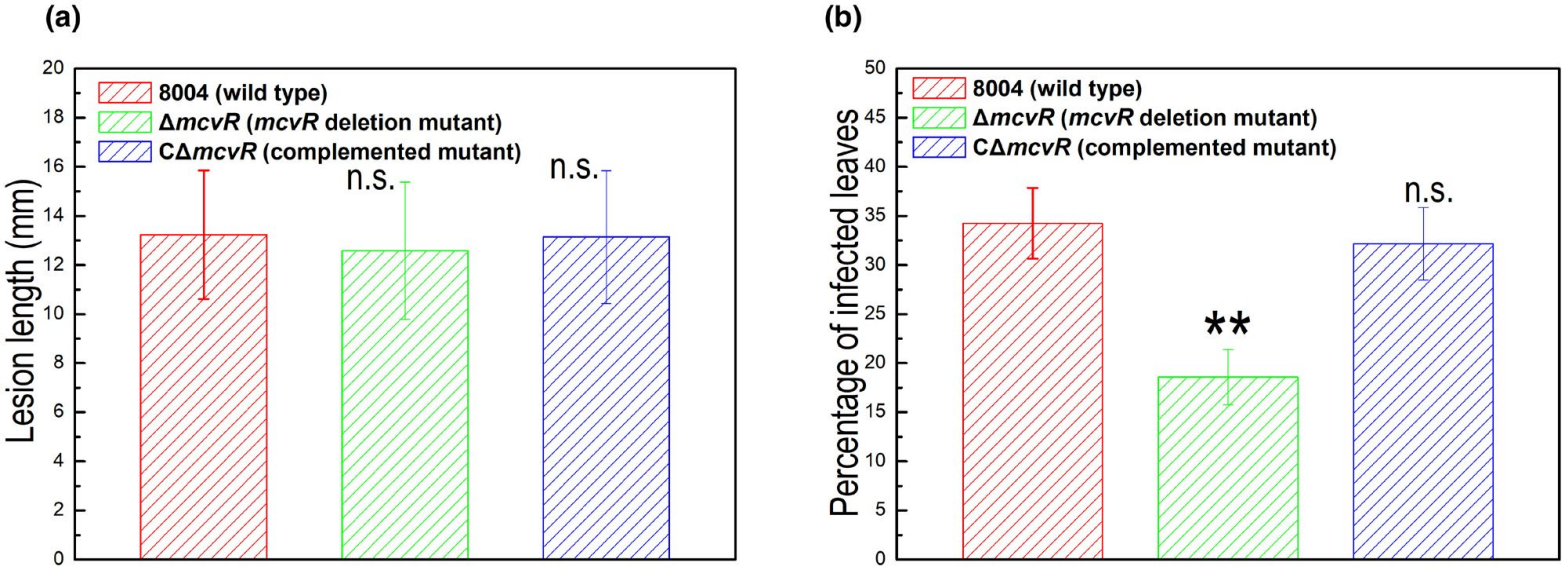
Effect of *mcvR* deletion on *Xanthomonas* *campestris* pv. *campestris* (Xcc) virulence. (a) The mutant showed normal virulence in host plants inoculated by leaf clipping. Xcc strains cells were resuspended in sterile distilled water at the concentration of 1 × 10^7^ CFU ml^−1^. Chinese radish (*Raphanus sativus*) leaves were cut with scissors dipped in the bacterial suspensions. Lesion lengths were scored at 10 days postinoculation. Values given are the mean and *SD* from 15 measurements in one experiment. Significance was determined by analysis of variance (ANOVA) and Dunnett's post hoc test for comparison to the wild type. The mean lesion length caused by the mutant was similar to the wild type. The experiment was repeated three times with similar results. (b) The mutant showed reduced virulence in host plants inoculated by the spraying method. A volume of 50 ml of the bacterial suspension (10^7^ cfu/ml) was sprayed onto the leaves of 25 plants (approximately 100 leaves). Three replicates of each independent experiment were carried out. Ten days after inoculation the relative virulence was determined as the percentage of the total inoculated leaves that showed the typical black rot disease. Values are means and *SD* of three replicates. Significance was determined by ANOVA and Dunnett's post hoc test for comparison to the wild type. **p < 0.01; n.s., not significant. The percentage of diseased leaves seen in response to the mutant was significantly smaller than in response to the wild type. Similar results were obtained in two other independent experiments

Xcc is a pathogen that generally invades host plant leaves through hydathodes and wounds, and multiplies in vascular tissues. The above data indicate that McvR is involved in swimming motility and chemotaxis. To explore the role of swimming motility and chemotaxis on pathogenesis in Xcc, we first tested the virulence of the Xcc strains on Chinese radish (*Raphanus* *sativus*) by the leaf‐clipping method. As shown in Figure 3a, the mean lesion lengths caused by the Δ*mcvR* mutant were similar to that caused by the wild‐type strain, indicating that the virulence of the mutant is similar to the wild type. We further tested the virulence of Xcc strains using the spraying method. The results showed that at 10 days after inoculation, the number of leaves that developed black rot symptoms in response to the mutant was significantly lower than the number showing disease in response to the wild type (Figure 3b). The number of diseased leaves in response to the complemented strain CΔ*mcvR* was not significantly different from the percentage of diseased leaves caused by the wild‐type strain (Figure 3b).

Overall, our findings show that mutation of *mcvR* caused a reduction in virulence of Xcc to Chinese radish when assayed by leaf spraying but not by the leaf cutting method, suggesting a role for McvR in the early invasion phase of the disease cycle.

### 2.2 McvR does not interact with the flagellar motor switch complex

The bacterial flagellum is used for swimming in aqueous environments and also, in some organisms, for swarming across solid surfaces (Jarrell & McBride, 2008). In many motile bacteria, the direction of rotation of flagellum is controlled by a complex of proteins at the bottom of the basal body called the switch complex (Porter et al., 2011).

Many SD‐RRs, such as CheY and its homologs, have been shown to regulate motility by interactions with the protein FliM, the main component of the flagellar motor switch complex (Szurmant & Ordal, 2004). VemR in Xcc controls motility via binding to FliM. We therefore speculated that McvR might influence swimming (flagellum‐dependent) motility through interaction with the switch complex. To test this hypothesis, potential interactions of FliM (XC_2267) with McvR were tested by using the bacterial two‐hybrid method.

**FIGURE 4:**
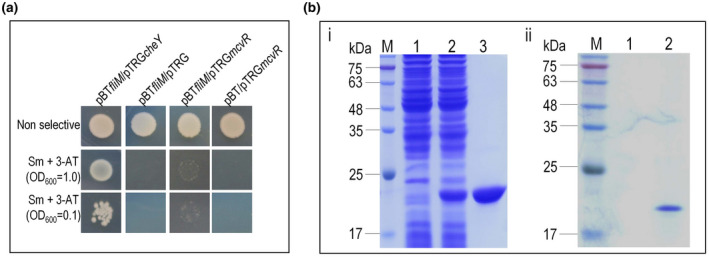
Interaction test between McvR and the flagellar motor protein FliM of *Xanthomonas campestris* pv. *campestris*. (a) Bacterial two‐hybrid experiment testing McvR–FliM interaction. CheY was used as a positive control. The reporter strain *Escherichia coli* XL1‐Blue MRF′ with different plasmid pairs was grown on nonselective plates (inoculated with a cell concentration of OD_600_ = 1.0) and double‐selection indicator plates (inoculated with cell concentrations of OD_600_ = 1.0 and 0.1) containing 3‐amino‐1,2,3‐triazole (3‐AT) and streptomycin (Sm). Protein–protein interactions activate the expression of *addA* and *his3* genes within the reporter gene cassette of the reporter strain, resulting in resistance to 3‐AT and Sm. The reporter strain with plasmid pair pBT*fliM*/pTRG*cheY* (Li et al., 2020) was able to grow on the selective plates, indicating an interaction between CheY and FliM. The reporter strain with plasmid pair pBTfliM/pTRG*mcvR* formed poor colonies on the selective plates. (b) Pull‐down assay testing McvR–FliM interaction. (i) SDS‐PAGE analysis of expression and purification of the recombinant 6 × His‐tagged fusion protein McvR. Lane 1, crude extract of *E. coli* BL21/pET‐30a‐McvR before induction with isopropyl β‐D‐thiogalactopyranoside (IPTG); lane 2, crude extract of *E. coli* BL21/pET‐30a‐McvR after induction with IPTG; lane 3, affinity‐purified 6 × His::McvR protein; M, molecular mass marker. (ii) Bait protein FliM was biotinylated and immobilized to streptavidin sepharose beads. The test protein (McvR or CheY) with acetyl phosphate (AcP) was mixed with the bait protein and incubated. After elution, samples were separated on 12% SDS‐PAGE and visualized by Coomassie blue staining. Lane 1, biotinylated FliM::6 × His was mixed with protein 6 × His::McvR; lane 2, pull‐down of 6 × His::CheY by FliM::6 × His; M, molecular mass marker

The 1011‐bp *fliM* gene was amplified by PCR and cloned into the bait vector pBT, generating the plasmid pBT*fliM* (Li et al., 2020). *cheY* was used as a control. Interactions were assessed in the *E*. *coli* reporter strain XL1‐Blue MRF′. Tests revealed that the plasmid pairs pBT*fliM* and pTRG*cheY* grew on the double‐selection indicator plate containing 3‐amino‐1,2,4‐triazole (3‐AT) and streptomycin (Figure 4a), However, the reporter strain containing the plasmid pairs pBT*fliM* and pTRG*mcvR* could not grow on the double‐selection indicator plate, indicating that interactions between FliM and McvR did not occur. A pull‐down biotinylated protein–protein assay was further carried out to ascertain this. CheY was used as a positive control. The recombinant 6 × His‐tagged proteins FliM and CheY were overproduced as previously described (Li et al., 2020). To obtain 6 × His‐tagged McvR, the 369‐bp *mcvR* coding sequence was cloned into the expression vector pET‐30a and overexpressed in *E. coli* cells (Figure 4b‐i). After purification, recombinant protein pull‐down assays were performed. The results show that the FliM protein captured CheY but not McvR (Figure 4b‐ii).

### 2.3 McvR is a global regulator that affects the expression of genes involved in motility, chemotaxis, and various cellular processes in Xcc

The data from the bacterial two‐hybrid and pull‐down assays implied that McvR regulates motility in a manner that differs from the action of CheY. To get a better understanding of the function of McvR in Xcc, transcriptome analysis was conducted by RNA‐sequencing. For this, the Δ*mcvR* mutant and wild‐type strain 8004 were grown to mid‐exponential phase (OD_600_ = 0.6) in NYG medium. Total RNA was extracted from two independent biological replicates.

Out of the 4273 annotated protein‐coding genes in the genome of Xcc 8004, 324 were found to be differentially expressed by two‐fold or more in the McvR mutant (Table S2). Of these, 108 were up‐regulated and 216 were down‐regulated (Table S2). To confirm the transcriptome changes, end‐point reverse transcription PCR (RT‐PCR) was used as a semiquantitative approach to analyse the relative expression levels of several selected genes. Expression of these selected genes was consistent with the data from the transcriptome analyses (Table S3).

**FIGURE 5:**
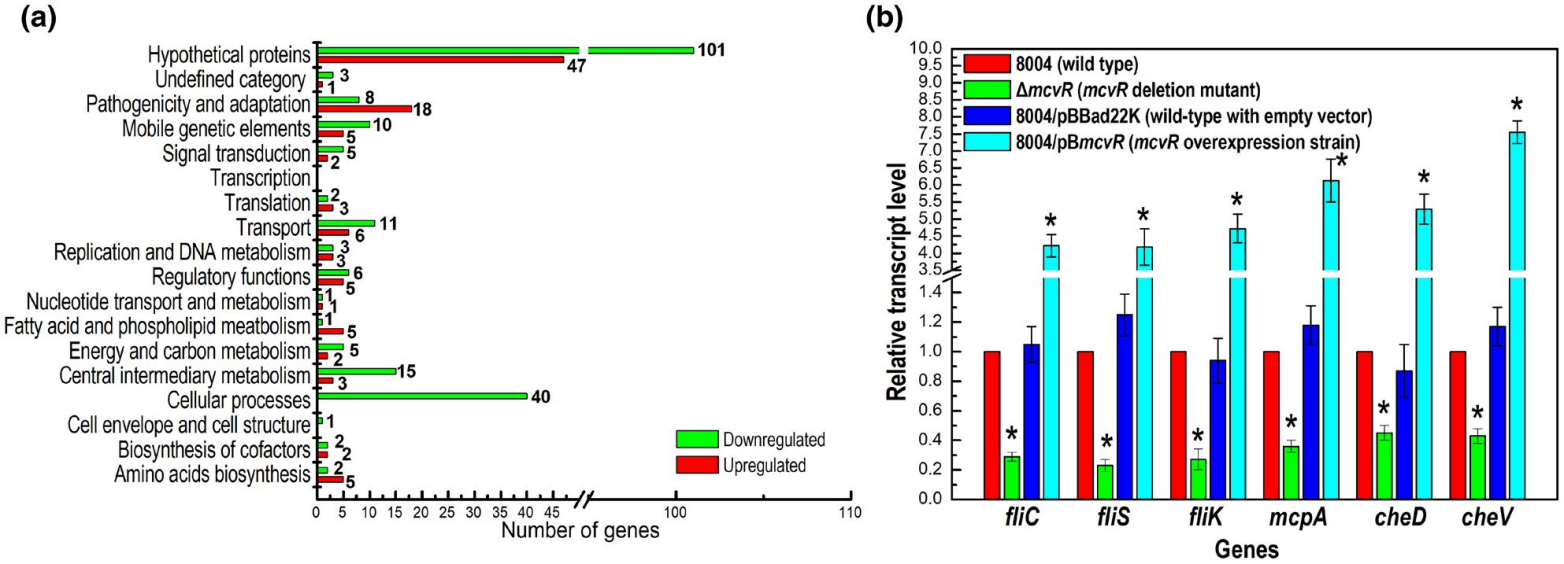
McvR affects the expression of numerous genes in *Xanthomonas campestris* pv. *campestris* (Xcc). (a) Functional categories of differentially expressed genes in the Δ*mcvR* mutant. Genome‐scale transcriptome profiling of Xcc strains cultured in NYG medium, which is widely used in studies of the morphology and biology of Xcc, were investigated by RNA‐sequencing and 324 genes were found to be differentially expressed by 2‐fold or more in the Δ*mcvR* mutant (Table S2). Basing on COG, 172 genes were assigned to 15 functional categories (He et al., 2007; Qian et al., 2005). (b) McvR positively regulates the expression of motility and chemotaxis‐related genes. Reverse transcription quantitative real‐time PCR (RT‐qPCR) analysis of the expression of selected genes in Xcc strains. RNA was isolated from cultures of Xcc strains grown in NYG medium for 24 h. The relative mRNA level was calculated with respect to the level of the corresponding transcript in the wild‐type strain 8004 (equal to 1). Values given are the means ± *SD* of triplicate measurements from a representative experiment; genes were considered to be differentially expressed if |log2 (fold change)| ≥ 1 compared to the wild type (*, significant). Similar results were obtained in two other independent experiments. The transcription level of the selected genes in the deletion mutant strain Δ*mcvR* and the overexpression strain 8004/pB*mcvR* was reduced and enhanced, respectively, compared to the wild type

Functional clustering analysis, according to the annotation of the Xcc 8004 genome, was carried out. A total of 172 genes were assigned to 15 functional categories that are based on cluster of genes (COG), but the remaining 152 genes encoded hypothetical proteins or have not been given a functional category to date (Figure 5a, Table S2) (He et al., 2007; Qian et al., 2005).

A total of 40 genes were identified belonging to the group of “cellular processes”, 26 to “pathogenicity and adaptation”, 18 to “central intermediary metabolism”, and 17 to “transport” (Figure 5a; Table S2). Notably, the deletion of *mcvR* had a significant negative impact on genes that contribute to flagella‐dependent motility (swimming) and chemotaxis (Table S2). For example, seven genes (*flgM*, *fliC*, *fliD*, *fliS*, *fliI*, *fliJ*, and *fliK*) that encode the proteins involved in flagellum synthesis and 32 genes that encode proteins involved in chemotaxis (Table S2) were down‐regulated in the Δ*mcvR* deletion mutant. To verify the regulation of McvR on motility and chemotaxis via controlling the expression of the flagellum synthesis and chemotaxis‐related genes, several related genes (*fliC*/*XC_2245*, *fliS*/*XC_2247*, *fliK*/*XC_2265*, *mcpA*/*XC_2504*, *cheD*/*XC_2322*, and *cheV/XC_2233*) were selected to quantify their expression levels. This was achieved by using reverse transcription quantitative real‐time PCR (RT‐qPCR) assays, in which we compared RNA amounts of the tested genes between the wild type and the Δ*mcvR* mutant cultured in NYG medium (Figure 5b). The results demonstrated that transcript levels for all the tested genes were reduced in the Δ*mcvR* mutant compared to the wild type.

Taken together, the findings suggest that, under the conditions tested, McvR has a broad regulatory role in Xcc.

### 2.4 Overexpression of *mcvR* enhances cell motility, chemotaxis, and virulence

The above data demonstrated that mutation in *mcvR* reduces virulence, swimming motility, and chemotaxis in Xcc. To get more knowledge about the function of the McvR protein, we further tested the effects caused by overexpression of McvR. To do this, the 369‐bp *mcvR* gene was amplified from Xcc genomic DNA by PCR and cloned into pBBad22K (Sukchawalit et al., 1999) under the control of an arabinose‐inducible promoter (P_ara_), generating the construct pB*mcvR*. The pB*mcvR* construct was introduced into Xcc 8004 using triparental conjugation, resulting in strain 8004/pB*mcvR*. As a control, pBBad22K was also introduced into wild‐type strain 8004. The expression levels of *mcvR* in Xcc strains 8004, 8004/pBBad22K, and 8004/pB*mcvR* were assessed by RT‐qPCR. *mcvR* expression was elevated in 8004/pB*mcvR*, and the *mcvR* transcript level in the overexpressing strain was more than 4‐fold higher compared to the wild‐type strain carrying the empty vector pBBad22K grown in NYG, even without addition of arabinose (Figure S3). This is consistent with previous findings that P_ara_ can ensure the overexpression of a target gene in the absence of arabinose in Xcc cells (Tang et al., 2020).

To obtain further insight into the regulatory role of McvR on the expression of flagellum synthesis and chemotaxis‐related genes, the expression level of the five selected genes (*fliC*, *fliS*, *fliK*, *mcpA*, *cheD*, and *cheV*) was assessed by RT‐qPCR. The results demonstrated that the expression levels of these genes in the strain 8004/pB*mcvR* was enhanced compared to the control strain 8004/pBBad22K (Figure 5b), indicating that McvR positively regulates the expression of flagellum synthesis and chemotaxis‐related genes.

**FIGURE 6:**
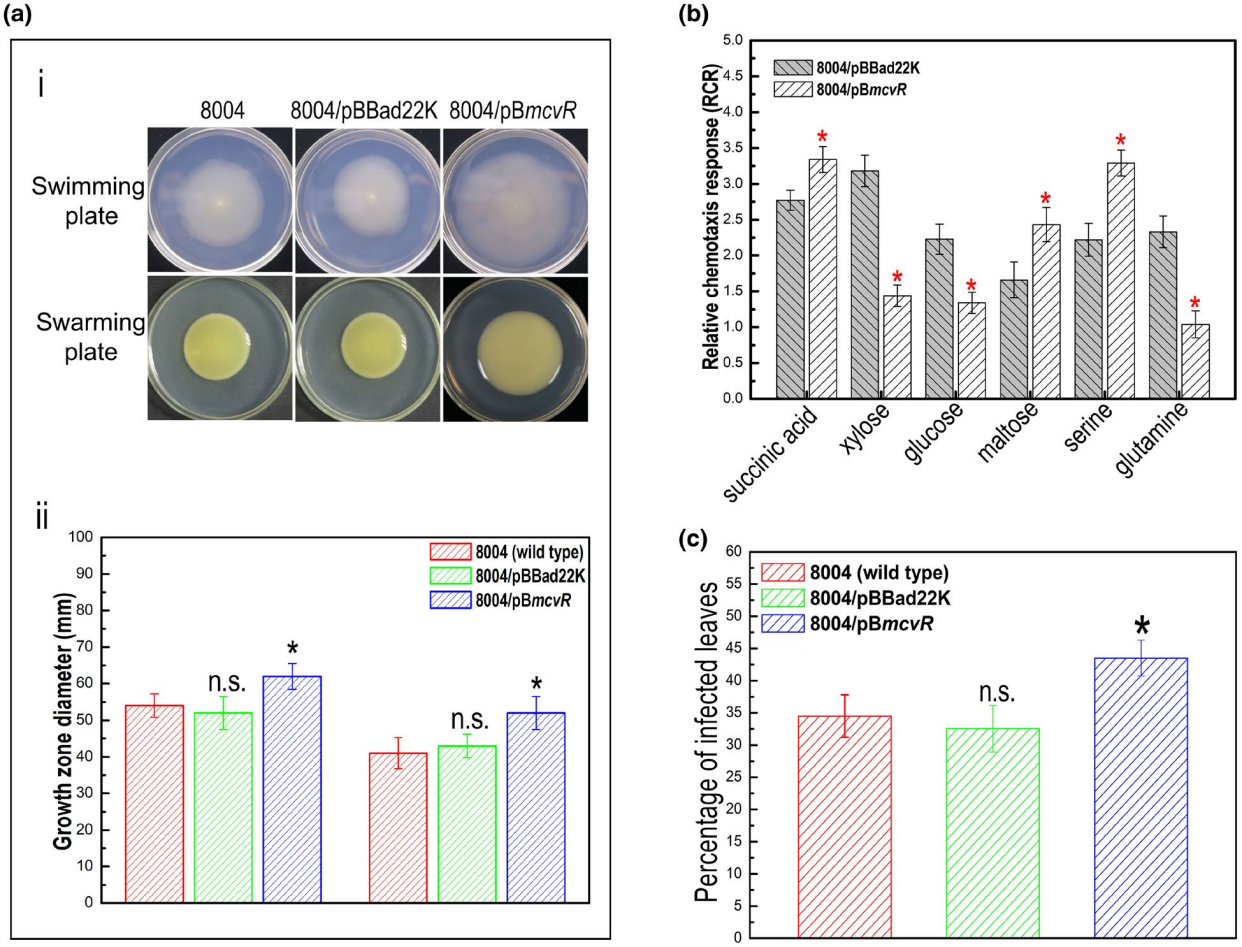
Overexpression of *mcvR* enhanced the cell motility, chemotaxis, and virulence in *Xanthomonas campestris* pv. *campestris* (Xcc). (a) Cell motility of different Xcc strains. An overnight culture of each Xcc strain was stabbed into “swim” (0.28% agar) medium or inoculated onto “swarm” (0.6% agar) medium. The representative colony morphologies of Xcc strains were photographed (i) and colony diameters of each strain on the different media were measured (ii). Values given are the mean ± *SD* of 10 measurements from a representative experiment. Significance was determined by analysis of variance (ANOVA) and Dunnett's post hoc test for comparison to the wild type. *p < 0.05; n.s., not significant. The experiment was repeated three times with similar results. (b) Relative chemotaxis response (RCR) of different Xcc strains. Several chemotactic agents, including succinic acid, xylose, glucose, maltose, serine, and glutamine, were selected to test the chemotaxis of Xcc strains 8004/pBBad22K and 8004/pB*mcvR*. The asterisks on each column indicate significantly different values compared with the value of the wild type with the empty vector (Student's *t* test; *p* < 0.05). Similar results were obtained in two other independent experiments. (c) Infection of different Xcc strains. Suspension of Xcc strains was sprayed onto the leaves of Chinese radish (*Raphanus sativus*). Three replicates of each independent experiment were carried out. Ten days after inoculation the percentage of the total number of inoculated leaves that showed the typical black rot disease symptoms was determined. Values are means and *SD* of three replicates. The percentage of infected leaves caused by the *mcvR*‐overpression strain is significantly larger compared to the wild type at *p* < 0.05 by ANOVA and Dunnett's post hoc test. Similar results were obtained in two other independent experiments

Xcc strains were tested for changes in cell motility, chemotaxis, extracellular enzymes, and EPS production. Differences were seen between the strains 8004/pB*mcvR* and 8004/pBBad22K in motility and chemotaxis (Figure 6a,b), but not the extracellular enzymes and EPS production (data not shown). Virulence of the *mcvR*‐overexpression strain 8004/pB*mcvR* was also estimated using the spraying method. As shown in Figure 6c, 8004/pB*mcvR* caused 43.5% of inoculated leaves to develop black rot symptoms, which was a significantly higher infection rate than the wild type (34.5%).

Taken together, the data show that McvR regulates motility and chemotaxis, as well as the expression of the flagellum synthesis and chemotaxis‐related genes in Xcc, suggesting that *mcvR* might influence motility and chemotaxis via the control of the expression level of the relevant genes.

### 2.5 McvR interacts with CheY and a subset of DNA‐binding proteins, including transcriptional regulators

Given that McvR is a SD‐RR, thus lacking the DNA‐binding domain, we supposed that McvR might cooperate with certain DNA‐binding proteins to perform a regulatory role in the process of regulating gene expression. To test this hypothesis, we first searched for McvR‐interacting proteins using co‐immunoprecipitation (co‐IP) coupled with liquid chromatography tandem‐mass spectrometry (LC‐MS/MS).

**FIGURE 7:**
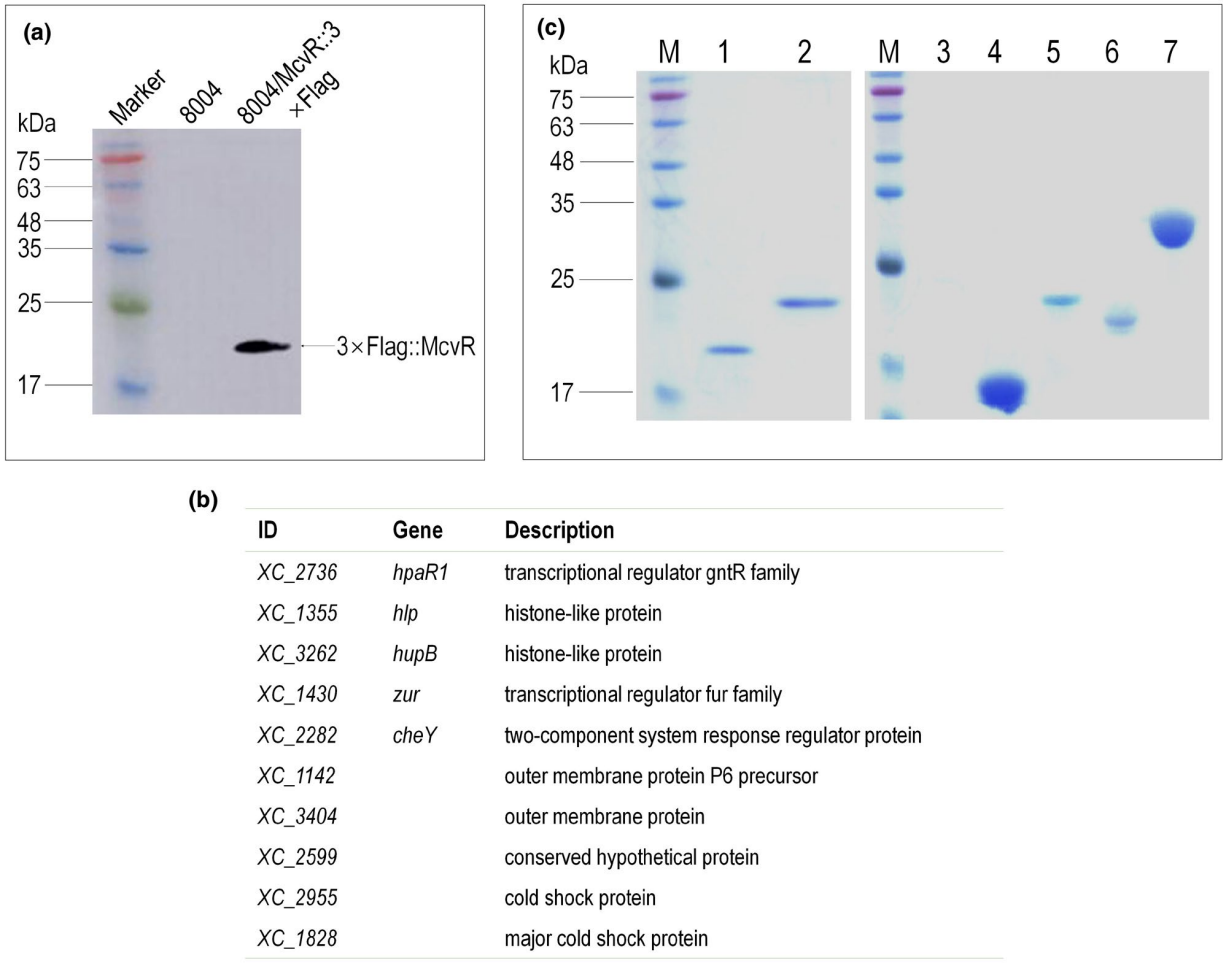
Identification of proteins that interact with the single‐domain response regulator McvR. (a) Western blot of the eluted McvR::3 × Flag fusion protein. After co‐immunoprecipitation (co‐IP), a western blot assay was performed for the eluted McvR::3 × Flag fusion protein and control. Protein samples were separated by SDS‐PAGE and transferred to a polyvinylidene difluoride (PVDF) membrane. The presence of the fusion proteins was detected by an anti‐Flag monoclonal antibody. (b) Candidate target proteins for McvR were identified by co‐IP combined with liquid chromatography tandem‐mass spectrometry. Experiments were repeated three times, and these candidate target proteins presented in all the three experiments. (c) McvR interactions confirmed by pull‐down assays. 6 × His‐tagged fusion proteins were overexpressed and purified. Bait protein McvR (or CheY) was biotinylated and immobilized to streptavidin sepharose beads. The potential prey protein CheY (or McvR), HpaR1, Hlp, HupB, or Zur was mixed with the bait protein and incubated. The FliM protein was used as control. After elution, samples were separated on 12% SDS‐PAGE and visualized by Coomassie blue staining. Lane 1, pull‐down of 6 × His::CheY by biotinylated 6 × His::McvR; lane 2, pull‐down of 6 × His::McvR by biotinylated 6 × His::CheY; lane 3, biotinylated 6 × His::McvR was mixed with protein FliM::6 × His; lane 4, pull‐down of 6 × His::HpaR1 by biotinylated 6 × His::McvR; lane 5, pull‐down of 6 × His::Hlp by biotinylated 6 × His::McvR; lane 6, pull‐down of 6 × His::Zur by biotinylated 6 × His::McvR; lane 7, pull‐down of 6 × His::HupB by biotinylated 6 × His::McvR; M, molecular mass marker

For these experiments strain 8004/McvR::3 × Flag (Table S1), expressing McvR fused to a 3 × Flag‐tag (McvR::3 × Flag), was constructed by in‐frame insertion of the 3 × Flag coding sequence into the 3ʹ end of the McvR‐coding sequence in the genome of Xcc 8004 (Table S1); Xcc wild‐type strain 8004 was used as a control. A western blot assay confirmed that the McvR::3 × Flag fusion protein could be eluted from strain 8004/McvR::3 × Flag, but not from the wild type (Figure 7a). Protein complexes with McvR::3 × Flag within Xcc cells were purified and analysed by LC‐MS/MS. The co‐IP coupled with LC‐MS/MS experiments were repeated three times, and selected the same identified proteins; proteins from the negative control were removed as the candidate McvR‐interacting proteins.

This analysis identified a set of 10 interacting proteins (Figure 7b). Notably, four proteins are known to have roles in DNA‐binding and/or regulation. XC_2736 (HpaR1) is a global regulator of diverse Xcc cellular processes (Su et al., 2016). XC_1430 (Zur), a key regulator of zinc homeostasis belonging to the Fur family of transcription factors, regulates the expression of a number of genes, including type III secretion system‐related genes (Huang et al., 2009). XC_1355 (Hlp) and XC_3262 (HupB), DNA‐binding proteins belonging to the HU family, play regulatory roles in virulence (Su et al., 2021). Interestingly, the central response regulator CheY in chemotaxis was also a potential partner protein of McvR.

A pull‐down biotinylated protein–protein assay was performed to validate the McvR interactions. Recombinant tagged proteins McvR, CheY, HpaR1, Zur, Hlp, and HupB were overexpressed in *E. coli*, and FliM was used as a negative control. After purification, recombinant protein pull‐down assays were performed. As shown in Figure 7c, McvR protein could capture CheY, HpaR1, Zur, Hlp, and HupB, but not FliM. Taken together, the data from the co‐IP and pull‐down assays indicate that McvR interacts with the response regulator CheY, suggesting that McvR can play its regulatory functions via association to CheY, as well as to other DNA‐binding proteins such as HpaR1, Zur, Hlp, and HupB.

### 2.6 The aspartate 55 is the phosphorylation site of McvR

According to background evidence from many SD‐RRs (Figure S4), the aspartyl residue at position 55 (Asp‐55) in McvR is likely to be the site of phosphorylation (Gao et al., 2019). To verify this prediction, we carried out an alanine substitution in McvR.

We substituted Asp‐55 in *mcvR* so that it encodes for alanine, inserting the resulting mutated sequence into the expression vector pET‐30a. Wild‐type McvR and the variant McvR_D55A_ were then overproduced and purified, and incubated with acetyl‐phosphate (AcP), which is known to phosphorylate the acceptor aspartyl residues of many response regulators. The results revealed that the wild‐type McvR could be phosphorylated by AcP. However, no phosphorylation of the variant McvR_D55A_ was observed, indicating that Asp‐55 is essential for phosphorylation.

**FIGURE 8:**
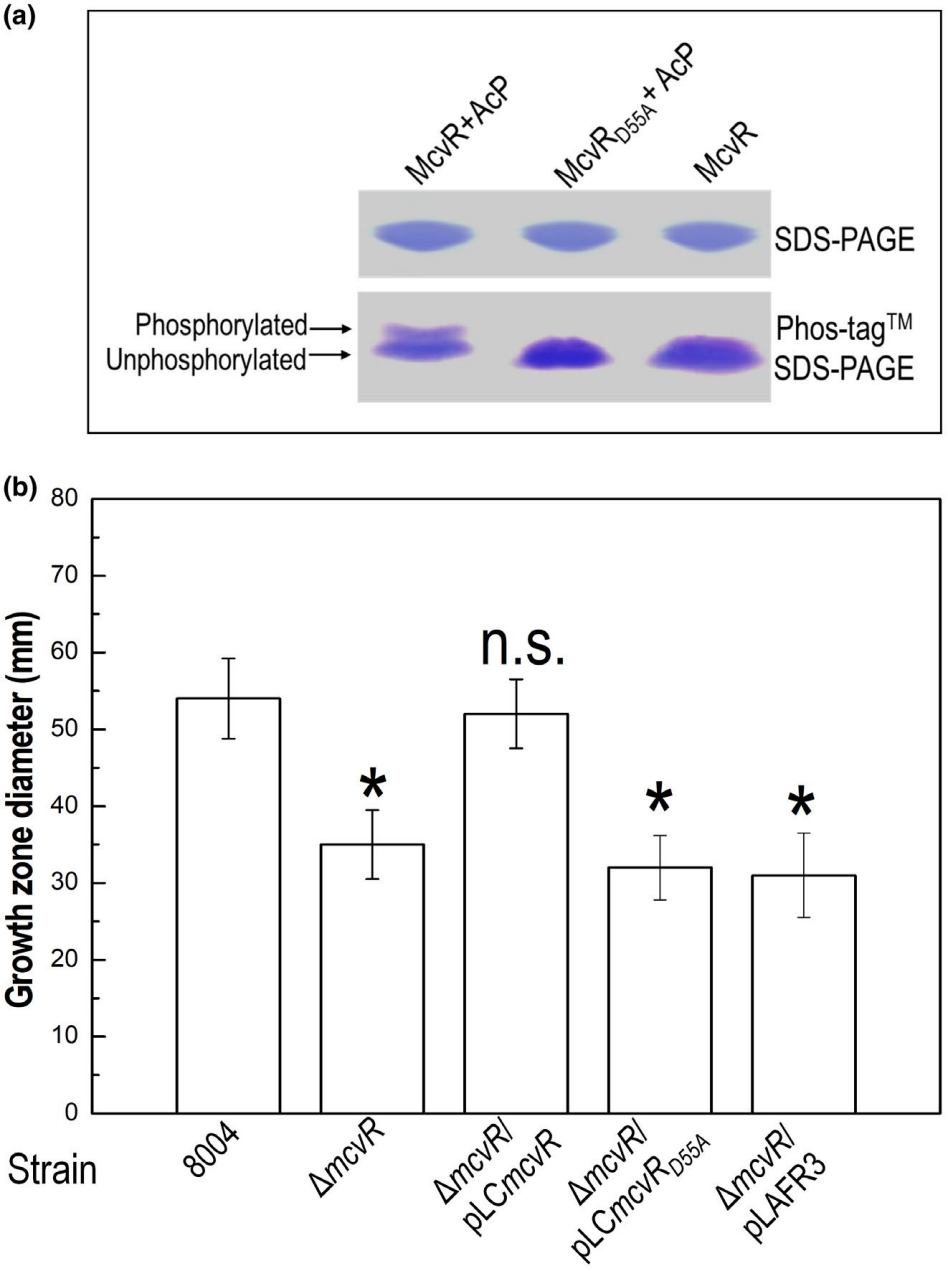
Aspartate 55 is the site of phosphorylation in McvR. (a) In vitro phosphorylation assays of McvR and its point mutants. Wild‐type McvR and its point mutants were incubated with acetyl‐phosphate (AcP), followed by detection on SDS‐PAGE and Phos‐tag^M^ SDS‐PAGE gels. The phosphorylated and unphosphorylated proteins were separated and could be seen on the Phos‐tag SDS‐PAGE gel. The wild‐type McvR could be phosphorylated by AcP, but not the variant McvR with aspartate substituted by alanine at position 55 (McvR_D55A_). Controls included a reaction containing only McvR and no phosphorylation by AcP was observed. (b) McvR with Asp 55 replacement lost its regulatory activity. *mcvR* mutant Δ*mcvR* was introduced with recombinant plasmids pLC*mcvR* and pLC*mcvR*_D55A_, or the empty vector pLAFR3. The resultant strains were tested for cell motility (swimming). *mcvR* carrying the altered code for Asla 55 did not restore the motility of the Δ*mcvR* mutant to the wild type level

The gene encoding the variant McvR protein with Asp‐55 replacement was further cloned into the plasmid pLAFR3, generating construct pLC*mcvR*_D55A_. This construct was introduced into the Δ*mcvR* mutant and the resulting strain was tested for cell motility. When pLC*mcvR*_D55A_ was introduced into Δ*mcvR*, no changes in swimming motility were seen (Figure 8b), indicating Asp‐55 is required for McvR regulatory activity.

## 3 DISCUSSION

The majority of characterized SD‐RRs, such as *E. coli* CheY or CheY‐like proteins in other bacteria, have been shown to regulate motility by intermolecular interactions with flagellar motor proteins FliM and FliN (Dyer & Dahlquist, 2006; Sarkar et al., 2010). Here we demonstrated that the SD‐RR McvR is involved in Xcc motility, chemotaxis, and virulence.

Chemotaxis is important for adaptation to changing environmental conditions in many bacteria. Bacteria sense surrounding environmental cues, such as sugars, amino acids, organic acids, inorganic salts, and chemical compounds, and the host environment via sensory chemotaxis systems and move via flagellum rotation to a favourable environment for their survival (Matilla & Krell, 2018). The *E. coli* CheY or CheY‐like proteins in other bacteria play a critical role in chemotaxis and motility, acting as a shuttle between the chemotaxis system and the flagella. During chemotaxis, bacteria sense chemical information and trigger a signalling cascade, which eventually leads to the phosphorylation of CheY (Parkinson et al., 2015). Phosphorylated CheY reverses the rotational direction of the flagellum via binding with higher affinity to the flagellar motor switch proteins FliM and FliN (Dyer & Dahlquist, 2006; Sarkar et al., 2010). Our bacterial two‐hybrid assay and pull‐down biotinylated protein–protein assays failed to detect a direct interaction between McvR and the flagellar motor protein FliM, suggesting that McvR must influence flagellum‐dependent motility in a different manner compared to CheY. Nevertheless, our co‐IP and pull‐down assays indicated that McvR does interact with CheY. Although the effect of CheY binding by McvR remains to be determined, we speculate that McvR might modulate the CheY–FliM interactions to control the rotation of the flagellar motor, thereby regulating behaviour.

Loss of McvR reduces the chemotaxis ability for certain chemotactic agents, swimming motility, and virulence in Xcc. Transcriptomic differential gene expression analysis and RT‐qPCR revealed that chemotaxis‐associated genes were down‐regulated in the *mcvR* mutant and up‐regulated in a *mcvR*‐overexpressing strain, indicating that McvR regulates chemotaxis‐ and motility‐related genes, and that McvR may promote chemotaxis ability and motility of Xcc by altering the expression of chemotaxis‐ and flagellar‐motility‐associated genes. Moreover, our transcription profiling data also revealed that McvR indirectly affected the expression of a number of genes. Currently, the mechanisms by which SD‐RRs may modulate gene expression are unclear. A further understanding of how McvR influences gene expression was explored using co‐IP coupled with LC‐MS/MS experiments and pull‐down assays. The results showed that McvR interacted with several DNA‐binding proteins such as HpaR1, Zur, Hlp, and HupB. These findings thus provide us with clues to how this protein is involved in the regulation of gene expression. We speculate that the affinity between McvR and different DNA‐binding proteins might be dependent on the phosphorylation state of McvR and that binding to them might modulate the DNA‐binding proteins’ regulatory properties. It is possible that McvR may act as an accessory element to CheY involved in modulating flagellar rotation in Xcc. A second possibility, not mutually exclusive, is that McvR regulates flagellar‐dependent motility and chemotaxis by controlling the expression of the series of motility‐ and chemotaxis‐related genes via interacting with certain DNA‐binding proteins.

The McvR protein harbours a receiver domain but possesses no output domains. Bioinformatic analysis suggests that the aspartyl residue at position 55 (Asp‐55) might be important for the functions of this protein. Our complementary test and in vitro phosphorylation assays demonstrated that the Asp‐55 is essential for controlling motility in Xcc. These data are consistent with *E. coli* CheY but do not rule out that part of its function may be executed via protein–protein interactions or by transferring phosphoryl groups between components in a phosphorelay such as the *B. subtilis* sporulation protein Spo0F, or draining phosphate away from histidine kinases such as *Rhizobium meliloti* CheY1 and *C. crescentus* DivK (Paul et al., 2008; Sourjik & Schmitt, 1998; Tzeng et al., 1998).

Xcc possesses a single polar flagellum to swim in an aqueous environment and in the presence of low agar concentrations (≤0.3% wt/vol). Only a few studies have focused on the motility and chemotaxis of xanthomonads, and the importance of motility and chemotaxis in the virulence of Xcc is still unclear. Normally, Xcc grows epiphytically and then enters the host plant through hydathodes on the leaf margin, and spreads from there through the leaf xylem vessels (Ryan et al., 2011). Previous studies showed that Xcc does not use flagellum‐dependent motility once inside host plants, but rather another form of swarming or pilus‐dependent motility, responsible for the movement of the bacterium in the xylem (Kamoun & Kado, 1990; Luneau et al., 2022). However, mechanisms that drive the epiphytical bacterial cells to move inside the host through hydathodes or wounds remain unclear. Here we demonstrated that the virulence of the *mcvR* mutant was deficient compared to the wild type only when host plants were inoculated with leaf spraying, and not when infected using the leaf‐clipping method, which directly introduces the bacteria directly into plants via wounds. This suggests that flagellum‐dependent motility and chemotaxis might play a key role in the early stage of invasion in Xcc, mainly required for the ingress into the host through hydathodes. Movement of the bacteria towards the plant openings is potentially mediated through chemotaxis. Although the *mcvR* mutants exhibited decreased or increased chemotaxis ability to different compounds, the agents that play key roles in plant entry, as well as the movement of bacterium in the epiphytic environment, remain to be identified.

Besides swimming motility, Xcc exhibits swarming motility on semisolid surfaces (0.5–0.7% agar) (Dunger et al., 2016). Even though deletion of *mcvR* had no effect on swarming, overexpression of *mcvR* did enhance this type of motility, indicating that McvR might positively regulate swarming in Xcc. This type of motility is usually defined as a rapid and coordinated multicellular movement of bacteria across a surface, propelled by rotating flagella, either by pulling via type IV pili (T4P) or by pushing with the secretion of slime (Kearns, 2010). Although the mechanism of swarming in Xcc is still unclear, T4P‐deficient mutants display reduced swarming in Xcc (Dunger et al., 2016), implying that swarming is a pilus‐associated type of motility. The mechanism by which McvR enhances swarming is worthy of future study.

## 4 EXPERIMENTAL PROCEDURES

### 4.1 Bacterial strains, plasmids, and growth conditions

The bacterial strains and plasmids used in this study are listed in Table S1. *E. coli* strains were grown in Luria–Bertani medium (Miller, 1972) or M9 (67.8 g Na_2_HPO_4_, 30 g KH_2_PO_4_, 5 g NaCl, 10 g NH_4_Cl per litre) at 37°C. Xcc strains were grown at 28°C in NYG medium (Daniels et al., 1984b), NY medium (NYG medium but without glycerol), and minimal medium MMX (Daniels et al., 1984a). Antibiotics were added at the following concentrations when required: kanamycin (Kan) 25 μg/ml, rifampicin (Rif) 50 μg/ml, ampicillin (Amp) 100 μg/ml, spectinomycin (Spc) 50 μg/ml, gentamicin (Gm) 5 μg/ml, streptomycin (Sm) at 100 μg/ml, and tetracycline (Tet) 5 μg/ml for Xcc strains, and 15 μg/ml for *E. coli*.

### 4.2 DNA and RNA manipulations

DNA manipulations followed the procedures previously described (Sambrook et al., 1989). Conjugations between Xcc and *E. coli* strains were performed as previously described (Turner et al., 1985). The restriction endonucleases, T4 DNA ligase, and *Pfu* polymerase were provided by Promega (Shanghai). The total RNA was extracted from Xcc strains using total‐RNA extraction kit (Invitrogen) and cDNA generated using a cDNA synthesis kit (Invitrogen). For end‐point reverse transcription PCR, the obtained cDNA was diluted and used as template with selected primers for target genes (Table S4).

To assay the transcription level of certain genes, RT‐qPCR was carried out as previously described (Li et al., 2014) with minor modifications. Briefly, PCRs were performed in a real‐time PCR thermal cycler (Analytik jena qTOWER2.0; Jena). Reactions included ChamQ universal SYBR qPCR master mix (Vazyme), corresponding primers (Table S4), and cDNA templates. Reactions with no template were used as negative controls. The melting curve analysis was performed at temperatures ranging from 60 to 95°C by raising 0.5°C/s. The relative mRNA level was calculated with respect to the level of the corresponding transcript in the wild‐type strain 8004 (equalling 1). The expression level of the 16S rRNA gene was used as an internal standard. The RT‐qPCR tests were performed in triplicate.

### 4.3 Deletion mutant construction and complementation

In‐frame deletion mutant of *mcvR* (*XC_1966*) was constructed by the method described by Schäfer et al. (1994). Then, 200–500 bp upstream and downstream fragments of the *mcvR* coding region were amplified using the corresponding primers listed in Table S4. Primers were modified to give *Bam*HI‐, *Xba*I‐, or *Hin*dIII‐compatible ends. The two fragments were cloned together into the vector pK18*mobsacB* (Schäfer et al., 1994), the resulted recombinant plasmid pK18*mobsacBmcvR* was introduced into Xcc 8004 by triparental conjugation, and transconjugants were screened on selective agar plates containing 5% sucrose. The obtained mutant was named Δ*mcvR* (Table S1).

For complementation of the Δ*mcvR* mutant, a 369‐bp *mcvR*‐coding sequence was amplified by PCR from the total DNA of Xcc 8004 with the corresponding primers listed in Table S4. Primers were modified to give *Bam*HI‐ or *Hin*dIII‐compatible ends. The amplified fragment was cloned into the plasmid pLAFR3 **(**Staskawicz et al., 1987 **)**. The generated plasmid pLC*mcvR* (Table S1) was introduced into the Δ*mcvR* mutant by triparental conjugation, generating a complemented strain named CΔ*mcvR* or Δ*mcvR*/pLC*mcvR* (Table S1).

For overexpression of *mcvR* in Xcc, the 369‐bp DNA fragment of *mcvR*‐coding sequence amplified using the corresponding primers listed in Table S4 was cloned into the broad‐host‐range expression vector pBBad22K (Sukchawalit et al., 1999), obtaining recombinant plasmid pB*mcvR*. This recombinant plasmid was introduced into the Xcc‐8004, resulting in strain 8004/pB*mcvR* (Table S1).

### 4.4 Site‐directed mutagenesis

Site‐directed mutagenesis was carried out using a QuikChange II Site‐directed Mutagenesis kit (Stratagene). The gene *mcvR* was cloned into pK18*mob* (Schäfer et al., 1994) and amplified by the specific PCR with mutagenic oligonucleotides listed in Table S4 and the recombinant plasmid pK*mcvR* as template. Here amino acid substitution of the aspartyl residue at position 55 in McvR product was developed. The PCR products were digested with *Dpn*I and transformed into *E. coli* DH5a, resulting in the recombinant plasmid containing *mcvR* with mutated codon(s). The mutated *mcvR* gene was cloned into plasmid pLAFR3, resulting in recombinant plasmid pLC*mcvR*_D55A_ (Table S1). The obtained recombinant plasmid was used for the complementation test. To obtain the variant McvR protein (McvR_D55A_), the mutated *mcvR* gene was cloned into the expression vector pET‐30a, resulting in recombinant plasmid pET‐30a‐McvR_D55A_ (Table S1).

### 4.5 Bacterial two‐hybrid assay

The BacterioMatch II two‐hybrid system (Stratagene) was carried out as previously described (Li et al., 2020) to detect McvR–protein interactions. Briefly, the 1011‐bp *fliM* gene, which was obtained by PCR using the corresponding primers listed in Table S4, was cloned into the bait vector pBT, generating the plasmid pBT*fliM* (Li et al., 2020). The 369‐bp *mcvR* or 378‐bp *cheY* coding sequence was PCR‐amplified from Xcc strain and cloned into the target vector pTRG, resulting in plasmids pTRG*mcvR* (Table S1) or pTRG*cheY* (Li et al., 2020). The plasmid pairs were co‐transformed into the reporter strain *E. coli* XL1‐Blue MRF′. Cells of the resulting strains were harvested and resuspended in M9 medium and adjusted to concentrations of OD_600_ = 1.0 and 0.1 (for selective plates only). The bacterial suspension was spotted on the nonselective plates and double‐selective indicator plates containing 5 mM 3‐AT and 12.5 μg/ml Sm, and then incubated at 28°C for 24 h.

### 4.6 Overproduction and purification of proteins

To overproduce the 6 × His‐tagged form of McvR and HupB, the 369‐bp *mcvR* and 270‐bp *hupB* (*XC_3262*) coding sequences were PCR‐amplified from Xcc 8004 using the corresponding primers listed in Table S4. The obtained DNA fragments were cloned into the expression vector pET‐30a or pET‐32a to generate the recombinant plasmids pET‐30a‐McvR and pET‐32a‐HupB (Table S1). The recombinant plasmids pET‐30a‐McvR (or pET‐30a‐McvR_D55A_, producing the McvR variant McvR_D55A_) and pET‐32a‐HupB were transformed into *E. coli* BL21. The obtained strains were cultured and induced by isopropyl *β*‐d‐thiogalactopyranoside (IPTG), and then the cells were collected and the fusion proteins were purified using Ni‐NTA resin (Qiagen). To obtain the FliM (XC_2267), CheY (XC_2282), Hlp (XC_1355), Zur (XC_1430), and HpaR1(XC_2736) proteins *E. coli* strains BL21/pET‐32a‐FliM, BL21/pET‐30a‐CheY, BL21/pET‐30a‐Hlp, M15/pQE‐30a‐Zur, and JM109/pQE‐30‐2736 expressing FliM, CheY, Hlp, Zur, and HpaR1, respectively, fused with a 6 × His‐tag on its N‐terminus or C‐terminus (Table S1) were grown and induced by IPTG.

### 4.7 Protein pull‐down assay

Protein pull‐down assays were performed as previously described (Li et al., 2014), with the ProFound pull‐down biotinylated protein–protein interaction kit (Pierce). Briefly, the FliM fusion protein FliM::6 × His or *mcvR* fusion protein 6 × His::McvR was biotinylated with sulfo‐NHS‐LC‐biotin. Then 50 μl of the purified biotinylated FliM::6 × His or 6 × His::McvR (0.5 mg/ml) was incubated with 40 μl of streptavidin sepharose beads. After washing, beads were incubated with 100 μl of sample containing 50 μg suspected prey protein 6 × His::McvR (or FliM::6 × His, 6 × His::CheY, 6 × His::HpaR1, 6 × His::Hlp, 6 × His::Zur, 6 × His::HupB) at 4°C for at least 60 min, and then beads were washed and prey protein was eluted using 150 μl of elution buffer (pH 2.8). Finally, 20 μl of the eluted sample was eletrophoresed on 12% SDS‐PAGE gel and visualized by Coomassie blue staining.

### 4.8 Co‐IP and LC‐MS/MS analysis

Co‐IP to identify McvR‐interacting partners was performed as previously described (Lai et al., 2018), with minor modifications. An Xcc strain expressing the recombinant protein McvR::3 × Flag was first constructed. A 655‐bp DNA fragment, which was composed of 240‐bp DNA upstream of the *mcvR* start codon, 369‐bp McvR‐coding sequence and 43‐bp Flag‐coding sequence, was generated by PCR amplification using the genomic DNA of strain 8004 as template and the primer set L*mcvR*‐FlagF/R (Table S4). Simultaneously, a 574‐bp DNA fragment, which was composed of 43‐bp Flag‐coding sequence, the 3‐bp stop codon of *mcvR*, and 528‐bp downstream of the *mcvR* stop codon, was generated by PCR amplification using the genomic DNA of strain 8004 as template and the primer set R*mcvR*‐FlagF/R (Table S4). The two fragments were joined using overlap extension PCR, and the resulting recombinant fragment was cloned into the suicide plasmid pK18*mobsacB* (Table S1). The resulting recombinant plasmid named pK*mcvR*::*Flag* (Table S1) was introduced into Xcc 8004, and the transconjugants were screened on selective agar plates and confirmed by DNA sequencing. This constructed variant strain was named 8004/McvR::3 × Flag (Table S1).

The 8004/McvR::3 × Flag strain and 8004 wild‐type strain (used as negative control) were grown in the NYG medium overnight, and cells were collected, washed, and lysed. After 10 min of centrifugation, the supernatant was transferred into fresh tubes. For each sample, 50 μl of agarose‐conjugated anti‐Flag were added and incubated at 4°C for 3 h. The agarose beads were washed three times and protein eluted with 0.25 M glycine (pH 2.5). The eluted proteins were resolved by SDS‐PAGE and analysed by LC‐MS/MS.

LC‐MS/MS was performed on a Nano‐LC system (Easy nLC 1000; Thermo Fisher Scientific) combined with an LTQ‐Orbitrap Elite mass spectrometer. Electrospray ionization (ESI) positive ion scans in a mass range of 300–1800 *m*/*z* were recorded at a resolution of 70,000 followed by data‐dependent top 20 higher‐energy collisional dissociation (HCD) fragmentation at a resolution of 17,500. The raw data were processed and searched for matches in a FASTA file of protein sequences generated from Xcc (strain 8004) Uniprot protein database (4239 sequences, https://www.uniprot.org/taxonomy/314565) using Proteome Discoverer 2.1 (Sequest algorithm). Search parameters were specified as follows: digestion–trypsin with two missed cleavages allowed; carbamidomethylation of cysteines (+57.021 Da) as a fixed modification; oxidation of methionines (+15.995 Da) and N‐terminal protein acetylation (+42.011 Da) as variable modifications. Mass tolerance of precursor ions was 12 ppm and that of fragment ions was ±0.02 Da. Only peptides that were filtered with a confidence level of 99% were accepted. The FDR for peptide was set to 1%. Co‐IP coupled with LC‐MS/MS was repeated three times, and proteins that were specifically precipitated by McvR in all the three experiments were considered to be the potential McvR‐interaction proteins.

### 4.9 Western blotting

Western blotting followed the procedure described by Sambrook et al. (1989). Bacterial proteins separated by SDS‐PAGE (or Phos‐tag SDS‐PAGE) gel were electrotransferred onto a PVDF membrane (Millipore). After blocking with 1% milk (or 5% bovine serum albumen), the proteins in the membrane were incubated with the 1:2500 diluted anti‐Flag‐tag (or anti‐His‐tag) mouse monoclonal antibody as the primary antibody, followed by washing four times with Tris‐buffered saline with Tween buffer (Tris 20 mM, NaCl 0.3 M, Tween 20 0.08% [vol/vol]). The diluted 1:2500 horseradish peroxidase‐conjugated goat anti‐mouse IgG (Bio‐Rad) was used as the secondary antibody. After washing the membrane four times, the luminescence signal was detected according to the manufacturer's instructions.

### 4.10 In vitro phosphorylation assay

In vitro phosphorylation assay was carried out as previously described (Li et al., 2020). First, 5 μg of the purified 6 × His::McvR (or its derivate 6 × His::McvR_D55A_) proteins were incubated with 50 mM lithium potassium acetyl phosphate (AcP) (Sigma) at 37°C for 30 min in a buffer (40 mM Tris‐HCl, pH 8.0, 10 mM MgCl_2_, 40 mM KCl, 1 mM dithiothreitol), then samples were separated and detected on SDS‐PAGE and Phos‐tag SDS‐PAGE gels.

### 4.11 Cell motility assays

Cell motility was tested as previous described (Su et al., 2016). To detect swimming motility, an overnight culture (OD_600_ = 1.0) of each Xcc strain was stabbed into 0.28% agar plates composed of 0.03% Bacto peptone and 0.03% yeast extract followed by incubation at 28℃ for 4 days. To test swarming motility, the bacterial cells were inoculated onto NY plates containing 2% glucose and 0.6% agar using a toothpick, and then incubated at 28℃ for 3 days. The diameter of the area occupied by strains was measured, and the values were used to indicate the motility of Xcc strains. The experiment was repeated three times.

### 4.12 Chemotaxis assays

Chemotaxis assays of Xcc strains were carried out using the syringe capillary method described previously (Mazumder et al., 1999; Pandey et al., 2016) with minor modifications. Xcc strains were grown in NYG medium overnight and diluted to OD_600_ = 0.6 (approximately 10^9^ cfu/ml), and then 100 μl of diluted culture was sucked into a disposable pipette tip and the chemotaxis capillary containing the chemoattractant was attached to bacterial suspension in a disposable pipette tip steadily at 28°C. Two hours later, the chemoattractant in the chemotaxis capillary was blown out and diluted to 10^5^ cfu/ml, and 100 μl diluted culture was plated onto NYG plate. Bacterial colonies were counted after incubating at 28°C for 3 days. Phosphate‐buffered saline was used as a control buffer for the test capillary. The relative chemotaxis fraction was calculated as cfu in test capillary versus cfu in control buffer capillary.

### 4.13 Plant assay

The virulence of Xcc to Chinese radish (*R. sativus*) was tested by the leaf‐clipping method (Dow et al., 2003) and the spraying method (An et al., 2014). Bacterial cells from overnight culture were collected, washed, and resuspended to a cell density of OD_600_ = 0.01 (approximately 10^7^ cfu/ml) in sterile distilled water. For the leaf‐clipping method, approximately 15 leaves were cut with scissors dipped in the bacterial suspensions for each independent experiment. Lesion length was measured 10 days after inoculation. For the spraying method, approximately 100 leaves were sprayed with a volume of 50 ml of the bacterial suspensions. Three replicates of each independent experiment were carried out. Ten days after inoculation the relative virulence was determined as the percentage of the total inoculated leaves that showed typical black rot disease symptoms at the leaf margin. The experiment was repeated three times.

### 4.14 Transcriptome analysis

Transcriptome analysis of the McvR deletion mutant Δ*mcvR* was performed as previously described (Cui et al., 2018). Briefly, Xcc strains were cultured in NYG medium to an OD_600_ = 0.6, and RNA was prepared. After the quantity determination and quality assessment, total RNA was sent to Novogene for library construction and strand‐specific RNA sequencing. Sequencing libraries were generated using a NEBNext Ultra Directional RNA Library Prep Kit for Illumina (New England BioLabs), and sequenced on an Illumina HiSeq 2000 platform. Clean reads were mapped to the reference genome and the RPKM (reads per kilobase per million mapped reads) method was used to calculate the gene expression levels. False discovery rate (FDR) ≤ 0.05 and |log_2_FC| (log_2_ of the fold changes) ≥ 1 were considered for differentially expressed genes. For confirmation, several differentially expressed genes were selected randomly to perform end‐point RT‐PCR analysis (Table S3).

## Supporting information


**FIGURE S1** Sequence alignments of McvR (XC_1966) with characterized single‐domain response regulators CheY (XC_2282) and VemR (XC_2252) in *Xanthomonas campestris* pv. *campestris*. Residues that are identical in two sequences and three sequences are highlighted with blue and red backgrounds, respectively

**FIGURE S2** Mutation in McvR has no impact on the extracellular polysaccharide (EPS) production and activity of extracellular enzymes in *Xanthomonas campestris* pv. *campestris* (Xcc). Plate assays were used to test the EPS production (a) and the activity of extracellular enzymes (b–d). An overnight culture (2 μl, OD_600_ = 1.0) of each Xcc strain was spotted onto a tested plate. For EPS production, bacteria on NY plates containing 2.0% (wt/vol) glucose were incubated at 28°С for 5 days. The *mcvR* mutant strain displayed similar colonies to the wild‐type strain 8004, indicating the EPS yield of the Δ*mcvR* strain was similar to that of the wild type. For estimation of the activity of extracellular enzymes, strains on NYG plates containing 0.5% (wt/vol) skim milk (for protease), 0.25% (wt/vol) carboxymethylcellulose (for endoglucanase) or 0.1% (wt/vol) starch (for amylase) were incubated at 28°С for 24 h (endoglucanase and amylase) or 48 h (protease). Plates were stained when necessary. Zones of clearance around the spot, due to the degradation of the substrate, from the Δ*mcvR* strain were similar to the wild‐type strain 8004, indicating the activity of extracellular enzymes of the *mcvR* mutant strain was similar to that of the wild type. Similar results were obtained in two other independent experiments

**FIGURE S3** Reverse transcription quantitative real‐time PCR (RT‐qPCR) assay to measure the transcription level of *mcvR* in *Xanthomonas campestris* pv. *campestris* (Xcc) strains 8004, 8004/pBBad22K, and 8004/pB*mcvR*. RNAs were extracted from Xcc cells cultured in NYG medium or NYG medium supplied with arabinose (for 8004/pB*mcvR* strain). The synergy brand (SYBR) green‐labelled PCR fragments were amplified as previous described (Li et al., 2014). The relative mRNA level was calculated with respect to the level of the corresponding transcript in the wild‐type strain 8004 (equalling 1). The expression level of the 16S rRNA gene was used as an internal standard. The RT‐qPCR tests were performed in triplicate. Values given are the mean ± *SD* from triplicate measurements in a representative experiment. Genes were considered to be differentially expressed if |log_2_(fold change)| ≥ 1 compared to the wild type (*, significant). Similar results were obtained in two other independent experiments

**FIGURE S4** Sequence alignments of McvR with CheY proteins from other organisms indicate that D55 (aspartyl residue at position 55) is the putative phosphorylation site. Multiple alignment was performed using the ClustalX program. The GenBank accession numbers of the nine CheY proteins are as follows: CAA53764.1 from *Listeria monocytogenes*, AKL84402.1 from *Bacillus atrophaeus*, AAA23577.1 from *Escherichia coli*, ABN14285.1 from *Burkholderia glumae*, SUP21169.1 from *Vibrio alginolyticus*, AAN03368.1 from *Pseudomonas fluorescens*, AAD01682.1 from *Helicobacter pylori*, CRX67012.1 from *Stenotrophomonas maltophilia*, AAM82681.1 from S*ynechococcus elongates*. Asterisk indicates the predicted phosphorylation site in these single‐domain response regulators

**TABLE S1** Strains and plasmids used in this study

**TABLE S2** The >2‐fold differentially expressed genes of the *mcvR*‐mutant strain cultured in NYG medium

**TABLE S3** Confirmation of RNA‐Seq gene expression level by end‐point reverse transcription‐PCR

**TABLE S4** Sequence of the primers used in this study

## Data Availability

The data that support the findings of this study are available from the corresponding author upon reasonable request.
