# Supplementary figures and images for: McvR, a single domain response regulator regulates motility and virulence in the plant pathogen Xanthomonas campestris
Source: Mol Plant Pathol. 2022 Feb 13;23(5):649–63. doi: 10.1111/mpp.13186 (PMC8995066; doi:10.1111/mpp.13186)

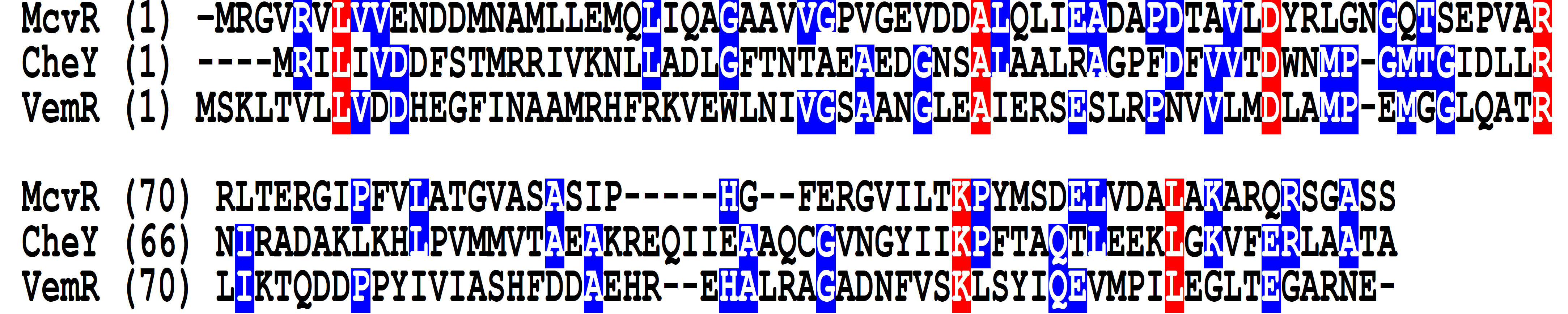

Supplement: Supplementary file 1 — FIGURE S1 Sequence alignments of McvR (XC_1966) with characterized single‐domain response regulators CheY (XC_2282) and VemR (XC_2252) in Xanthomonas campestris pv. campestris. Residues that are identical in two sequences and three sequences are highlighted with blue and red backgrounds, respectively [file MPP-23-649-s002.jpg]

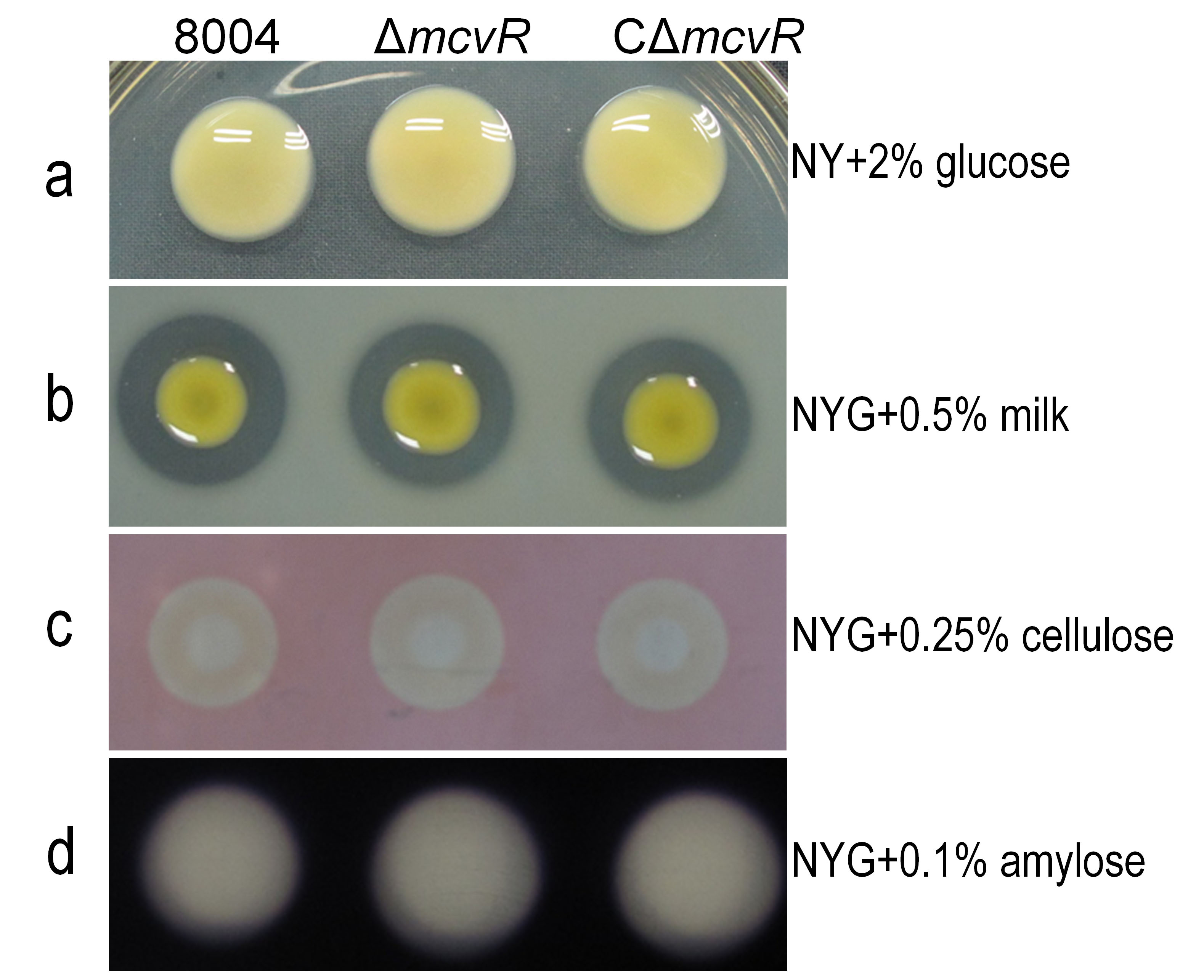

Supplement: Supplementary file 2 — FIGURE S2 Mutation in McvR has no impact on the extracellular polysaccharide (EPS) production and activity of extracellular enzymes in Xanthomonas campestris pv. campestris (Xcc). Plate assays were used to test the EPS production (a) and the activity of extracellular enzymes (b–d). An overnight culture (2 μl, OD600 = 1.0) of each Xcc strain was spotted onto a tested plate. For EPS production, bacteria on NY plates containing 2.0% (wt/vol) glucose were incubated at 28°С for 5 days. The mcvR mutant strain displayed similar colonies to the wild‐type strain 8004, indicating the EPS yield of the ΔmcvR strain was similar to that of the wild type. For estimation of the activity of extracellular enzymes, strains on NYG plates containing 0.5% (wt/vol) skim milk (for protease), 0.25% (wt/vol) carboxymethylcellulose (for endoglucanase) or 0.1% (wt/vol) starch (for amylase) were incubated at 28°С for 24 h (endoglucanase and amylase) or 48 h (protease). Plates were stained when necessary. Zones of clearance around the spot, due to the degradation of the substrate, from the ΔmcvR strain were similar to the wild‐type strain 8004, indicating the activity of extracellular enzymes of the mcvR mutant strain was similar to that of the wild type. Similar results were obtained in two other independent experiments [file MPP-23-649-s008.jpg]

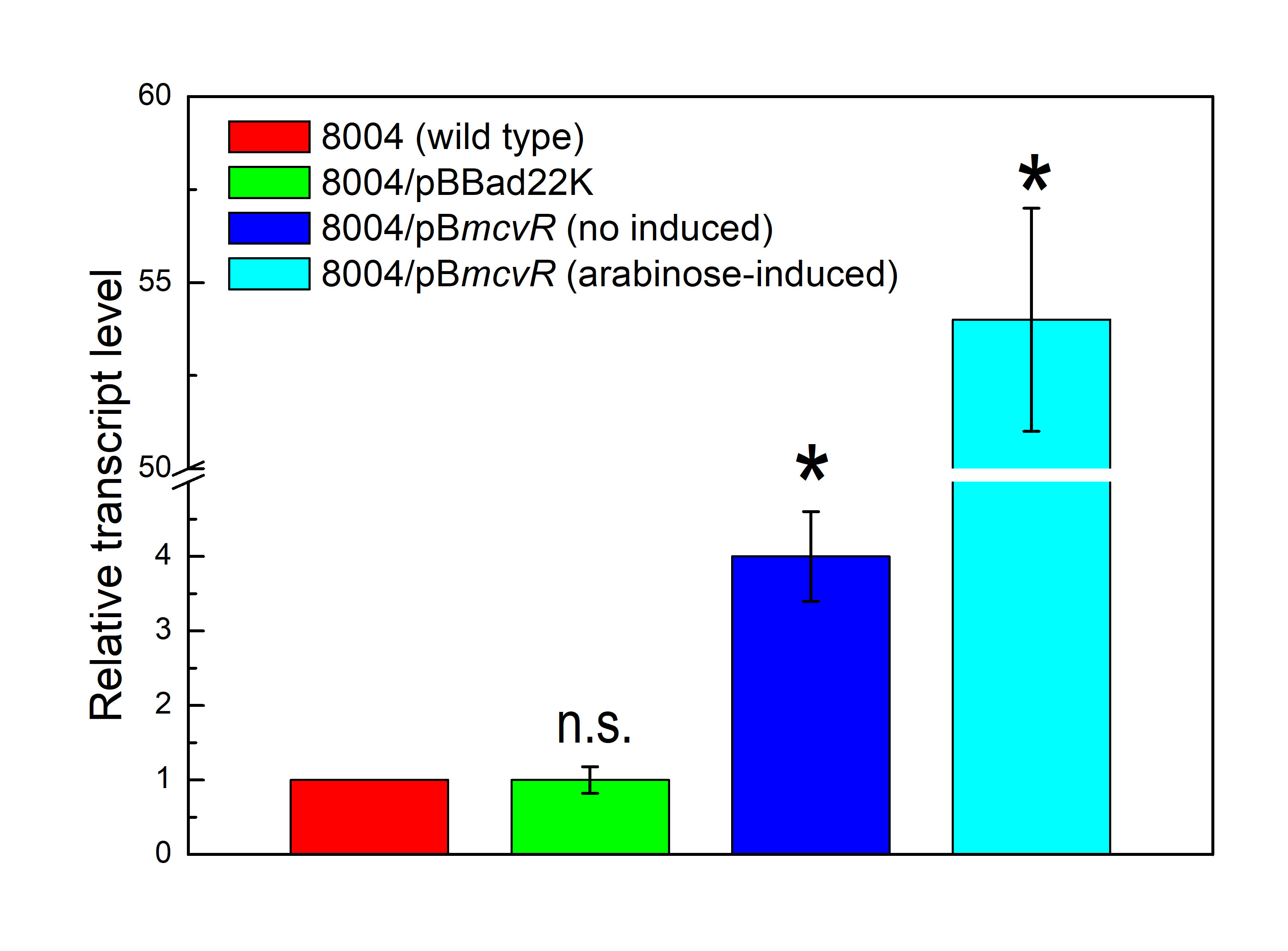

Supplement: Supplementary file 3 — FIGURE S3 Reverse transcription quantitative real‐time PCR (RT‐qPCR) assay to measure the transcription level of mcvR in Xanthomonas campestris pv. campestris (Xcc) strains 8004, 8004/pBBad22K, and 8004/pBmcvR. RNAs were extracted from Xcc cells cultured in NYG medium or NYG medium supplied with arabinose (for 8004/pBmcvR strain). The synergy brand (SYBR) green‐labelled PCR fragments were amplified as previous described (Li et al., 2014). The relative mRNA level was calculated with respect to the level of the corresponding transcript in the wild‐type strain 8004 (equalling 1). The expression level of the 16S rRNA gene was used as an internal standard. The RT‐qPCR tests were performed in triplicate. Values given are the mean ± SD from triplicate measurements in a representative experiment. Genes were considered to be differentially expressed if |log2(fold change)| ≥ 1 compared to the wild type (*, significant). Similar results were obtained in two other independent experiments [file MPP-23-649-s007.jpg]

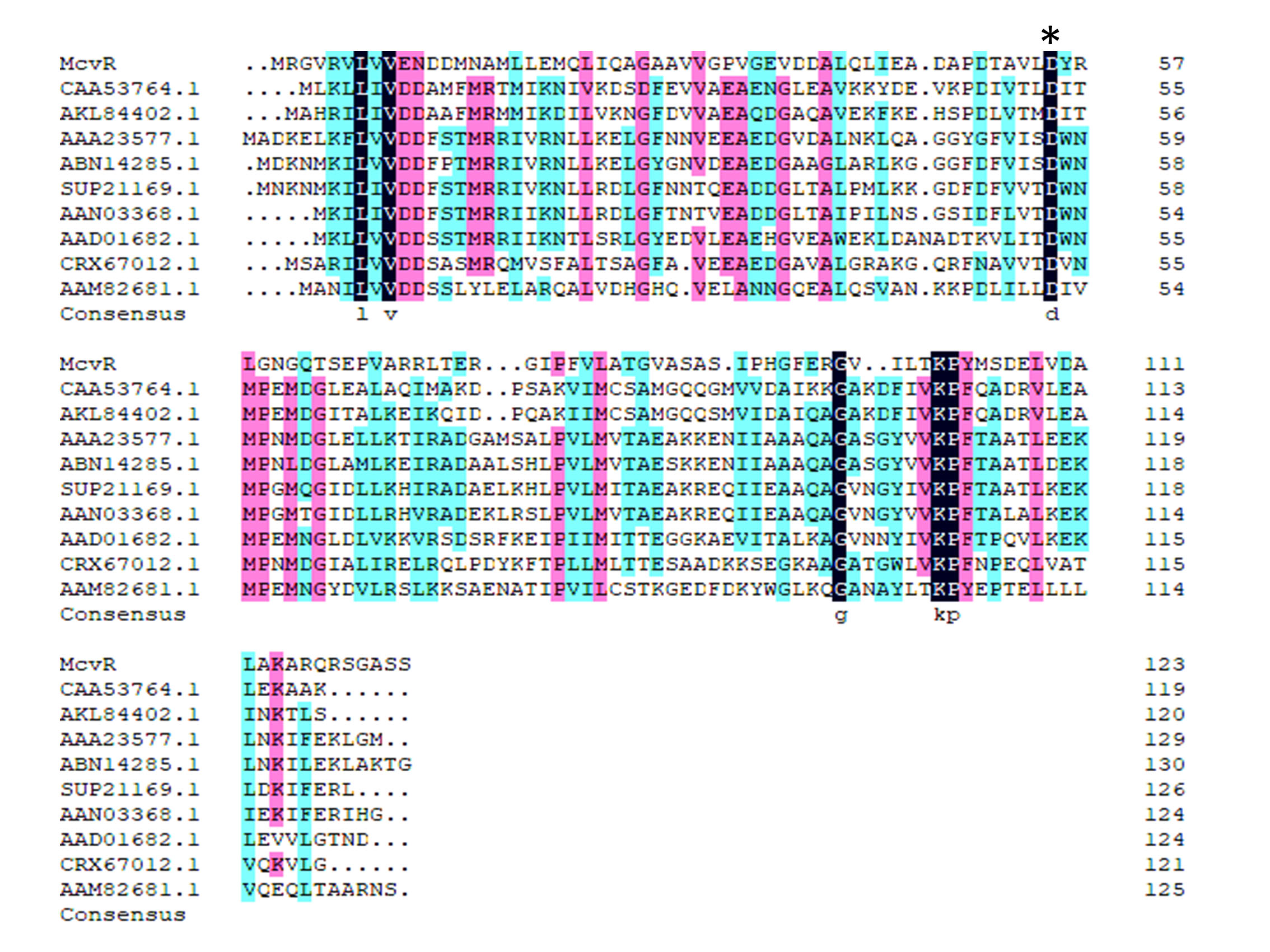

Supplement: Supplementary file 4 — FIGURE S4 Sequence alignments of McvR with CheY proteins from other organisms indicate that D55 (aspartyl residue at position 55) is the putative phosphorylation site. Multiple alignment was performed using the ClustalX program. The GenBank accession numbers of the nine CheY proteins are as follows: CAA53764.1 from Listeria monocytogenes, AKL84402.1 from Bacillus atrophaeus, AAA23577.1 from Escherichia coli, ABN14285.1 from Burkholderia glumae, SUP21169.1 from Vibrio alginolyticus, AAN03368.1 from Pseudomonas fluorescens, AAD01682.1 from Helicobacter pylori, CRX67012.1 from Stenotrophomonas maltophilia, AAM82681.1 from Synechococcus elongates. Asterisk indicates the predicted phosphorylation site in these single‐domain response regulators [file MPP-23-649-s005.jpg]
