# Supplementary material for: McvR, a single domain response regulator regulates motility and virulence in the plant pathogen Xanthomonas campestris
Source: Mol Plant Pathol. 2022 Feb 13;23(5):649–63. doi: 10.1111/mpp.13186 (PMC8995066; doi:10.1111/mpp.13186)
Supplement: Supplementary file 5 — TABLE S1 Strains and plasmids used in this study [file MPP-23-649-s006.docx]

**Table S1. Strains and plasmids used in this study**

| Strains or plasmids | Relevant characteristics | Reference or source |
| --- | --- | --- |
| *E. coli* strains |  |  |
| DH5α | Φ80△*lacZM*15 *recA1 endA1 deoR* | Gibco BRL, Life Technologies |
| M15 | *lac ara gal mtl recA1 uvr1* [pREP4 *lacI* Kan^r^] | Qiagen |
| JM109 | *RecA*1*, endA*1*, gyrA*96*, thi, supE*44*, relA*1  △ (*lac-proAB*)/F’ [*traD36, lacI*^q^, *lacZ* △M15] | Yanisch-Perron *et al*., 1985 |
| BL21(DE3) | F^-^ *ompT gal dcm lon hsdS_B_* (*r^-^_B_ m^-^_B_*) λ(DE3) | Novagen, Germany |
| BL21/pET-30a-McvR | BL21(DE3) harboring plasmid pET-30a-McvR | This work |
| BL21/pET-30a-McvR_D55A_ | BL21(DE3) harboring plasmid pET-30a-McvR_D55A_ | This work |
| BL21/pET-30a-CheY | BL21(DE3) harboring plasmid pET-30a-CheY | Li *et al*., 2020 |
| BL21/pET-32a-FliM | BL21(DE3) harboring plasmid pET-32a-FliM | Li *et al*., 2020 |
| BL21/pET-32a-HupB | BL21(DE3) harboring plasmid pET-32a-HupB | This work |
| BL21/pET-30a-Hlp | BL21(DE3) harboring plasmid pET-30a-Hlp | Su *et al*., 2021 |
| M15/pQE-30a-Zur | M15 harboring plasmid pQE-30a-Zur | This work |
| JM109/pQE-30-2736 | M15 harboring plasmid pQE-30-2736 | An *et al*., 2011 |
| XL1-Blue MRF' | Reporter strain, Δ*(mcrA)183* Δ*(mcrCB-hsdSMR-mrr)173 endA1 hisB supE44 thi-1 recA1 gyrA96relA1 lac* [F*´ lacIq HIS3 aadA* Kan*^r^*] | Stratagene |
| XL1-Blue MRF'/pBT*fliM*/pTRG*cheY* | XL1-Blue MRF' harboring plasmids pBT*fliM* and pTRG*cheY* | Li *et al*., 2020 |
| XL1-Blue MRF'/pBT*fliM*/pTRG | XL1-Blue MRF' harboring plasmids pBT*fliM* and pTRG | This work |
| XL1-Blue MRF'/pBT*fliM*/pTRG*mcvR* | XL1-Blue MRF' harboring plasmids pBT*fliM* and pTRG*mcvR* | This work |
| XL1-Blue MRF'/pBT/pTRG*mcvR* | XL1-Blue MRF' harboring plasmids pBT and pTRG*mcvR* | This work |
| *Xanthomonas campestris* pv. *campestris* strains |  |  |
| 8004 | Wild-type strain. Rif^r^ | Daniels *et al*., 1984 |
| 8004/pBBad22K | 8004 harboring an empty vector pBBad22K. Rif^r^ Km^r^ | This work |
| 8004/pB*mcvR* | 8004 harboring a recombinant plasmid pB*mcvR*. Rif^r^ Km^r^ | This work |
| Δ*mcvR* | As 8004, but *mcvR* gene (*XC_1966*) deleted. Rif^r^ | This work |
| C∆*mcvR* or Δ*mcvR*/pLC*mcvR* | Δ*mcvR* harboring a recombinant plasmid pLC*mcvR* derived from the full length of *mcvR* cloned into the plasmid pLAFR3. Rif^r^ Tet^r^ | This work |
| Δ*mcvR*/pLC*mcvR_D55A_* | Δ*mcvR* harboring a recombinant plasmid pLC*mcvR_D55A_*. Rif^r^ Tet^r^ | This work |
| Δ*mcvR*/pLAFR3 | Δ*mcvR* harboring an empty vector pLRAFR3. Rif^r^ Tet^r^ | This work |
| 8004/McvR::3×Flag | As 8004, but expresses the recombinant protein McvR::3×Flag. Rif^r^ | This work |
| Plasmids |  |  |
| pLAFR3 | Broad host range cloning vector, Tet^r^ | Staskawicz *et al*., 1987 |
| pK18*mob* | pUC18 derivative, *lacZα*, Kan^r^, *mob* site. Suicide plasmid in *Xcc*. | Schäfer *et al*. 1994 |
| pK*mcvR* | The suicide plasmid pK18*mob* containing 369-bp *mcvR* coding sequence of *Xcc* strain. Used for site-directed mutagenesis. Kan^r^ | This work |
| pK18*mobsacB* | pUC18 derivative, *lacZα*, *sacB*, Kan^r^, *mob* site. Allelic exchange vector (Suicidal vector carrying *sacB* gene for mutagenesis) | Schäfer *et al*., 1994 |
| pK18*mobsacBmcvR* | pK18*mobsacB* containing fragments flanking *mcvR*. Kan^r^ | This work |
| pK*mcvR*::*flag* | pK18*mobsacB* containing fragment composing 240-bp upstream of the *mcvR*, 369-bp McvR-coding sequence, 66-bp 3×Flag-coding sequence, 3-bp stop codon and 528-bp downstream of the *mcvR*. | This work |
| pLC*mcvR* | 369-bp DNA fragment of *Xcc mcvR* ORF cloned into the plasmid pLAFR3. Tet^r^ | This work |
| pLC*mcvR_D55A_* | 369-bp DNA fragment of *Xcc* *mcvR* ORF in which the aspartate at position 55 was replaced by alanine cloned into the plasmid pLAFR3. Tet^r^ | This work |
| pET-30a | Expression vector, allow the production of fusion proteins containing amino terminal 6×His-tagged sequences. Kanr | Novagen |
| pET-30a -McvR | pET-30a containing a 369-bp fragment of *mcvR* gene coding region | This work |
| pET-30a-McvR_D55A_ | pET-30a containing a 369-bp fragment of point-mutated *mcvR* gene ( replacing aspartate at position 55 to alanine) | This work |
| pET-30a-CheY | pET-30a containing a 378-bp fragment of *cheY* gene coding region | Li *et al*., 2020 |
| pET-30a-Hlp | pET-30a containing a *hlp* gene coding sequence | Su *et al*., 2021 |
| pET-32a | Expression vector, allow the production of fusion proteins containing amino terminal thioredoxin-tagged and carboxyl-terminal 6×His-tagged sequences. Amp^r^ | Novagen |
| pET-32a-FliM | pET-30a containing a 1011-bp fragment of *fliM* (*XC_2267*) gene coding region | Li *et al*., 2020 |
| pET-32a-HupB | pET-32a containing a *hupB* gene coding sequence | This work |
| pQE-30a | Expression vector, allowing the production of fusion proteins containing amino terminal 6×His-tagged sequences. Ampr | Qiagen, Germany |
| pQE-30-2736 | pQE-30a containing a *hpaR1* gene coding sequence | An *et al*., 2011 |
| pQE-30a-Zur | pQE-30a containing a *zur* gene coding sequence | Authors lab collection |
| pBT | Two-hybrid system bait plasmid containing the *cat* gene, p15A origin of replication and λ cI ORF. | Stratagene |
| pBT*fliM* | pBT derivative carrying a 1011-bp fragment of *fliM* gene. Cat^r^ | Li *et al*., 2020 |
| pTRG | Two-hybrid system target plasmid containing the *tet* gene, ColE1 origin of replication, and RNA polymerase α subunit ORF. | Stratagene |
| pTRG*mcvR* | pTRG derivative carrying 369-bp fragment of *mcvR* gene coding region. Tet^r^ | This work |
| pTRG*cheY* | pTRG derivative carrying 378-bp fragment of *cheY* gene coding region. Tet^r^ | Li *et al*., 2020 |
| pBBad22K | Km^r^, L-arabinose-inducible broad host range vector based on the pBBR1MCS-4 replicon | Sukchawalit *et al*., 1999 |
| pB*mcvR* | pBBad22K containing 369-bp *mcvR* coding sequence. | This work |

**References**

An, S.Q., Lu, G.T., Su, H.Z., Li, R.F., He, Y.Q., Jiang, B.L., Tang, D.J., & Tang, J.L. (2011). Systematic mutagenesis of all predicted gntR genes in *Xanthomonas campestris* pv. *campestris* reveals a GntR family transcriptional regulator controlling hypersensitive response and virulence. *Molecular plant-microbe interactions*, *24*(9), 1027–1039.

Daniels, M.J., Barber, C.E., Turner, P.C., Sawczyc, M.K., Byrde, R.J.W., and Fielding, A.H. (1984) Cloning of genes involved in pathogenicity of *Xanthomonas campestris* pv. *campestris* using the broad host range cosmid pLAFR1. *EMBO J* **3:** 3323–3328.

Li, R.F., Lu, G.T., Li, L., Su, H.Z., Feng, G.F., Chen, Y., He, Y.Q., Jiang, B.L., Tang, D.J., & Tang, J.L. (2014). Identification of a putative cognate sensor kinase for the two-component response regulator HrpG, a key regulator controlling the expression of the *hrp* genes in *Xanthomonas campestris* pv. *campestris*. *Environmental microbiology*, *16*(7), 2053–2071.

Li, R.F., Wang, X.X., Wu, L., Huang, L., Qin, Q.J., Yao, J.L., Lu, G.T., & Tang, J.L. (2020). *Xanthomonas campestris* sensor kinase HpaS co-opts the orphan response regulator VemR to form a branched two-component system that regulates motility. *Molecular plant pathology*, *21*(3), 360–375.

Schäfer, A., Tauch, A., Jäger, W., Kalinowski, J., Thierbach, G., & Pühler, A. (1994). Small mobilizable multi-purpose cloning vectors derived from the *Escherichia coli* plasmids pK18 and pK19: selection of defined deletions in the chromosome of *Corynebacterium glutamicum*. *Gene*, *145*(1), 69–73.

Staskawicz, B., Dahlbeck, D., Keen, N., & Napoli, C. (1987). Molecular characterization of cloned avirulence genes from race 0 and race 1 of *Pseudomonas syringae* pv. *glycinea*. *Journal of bacteriology*, *169*(12), 5789–5794.

Su, Q., Wang, X.X., Leng, M., Qi, Y.H., Pang, F.Y., Tang, J.L., & Lu, G.T. (2021). A HU-like protein is required for full virulence in *Xanthomonas campestris* pv. *campestris*. Molecular plant pathology, 10.1111/mpp.13128. Advance online publication. https://doi.org/10.1111/mpp.13128

Sukchawalit, R., Vattanaviboon, P., Sallabhan, R., and Mongkolsuk, S. (1999) Construction and characterization of regulated L-arabinose-inducible broad host range expression vectors in *Xanthomonas*. *FEMS microbiology letters*, *181*(2), 217–223.

Yanisch-Perron, C., Vieira, J., & Messing, J. (1985). Improved M13 phage cloning vectors and host strains: nucleotide sequences of the M13mp18 and pUC19 vectors. *Gene*, *33*(1), 103–119.
