# Supplementary material for: McvR, a single domain response regulator regulates motility and virulence in the plant pathogen Xanthomonas campestris
Source: Mol Plant Pathol. 2022 Feb 13;23(5):649–63. doi: 10.1111/mpp.13186 (PMC8995066; doi:10.1111/mpp.13186)
Supplement: Supplementary file 6 — TABLE S2 The >2‐fold differentially expressed genes of the mcvR‐mutant strain cultured in NYG medium [file MPP-23-649-s003.doc]

**Table S2.** The >2-fold differentially expressed genes of the mcvR-mutant strain cultured in the NYG medium

| Function Category | Gene ID | Name | Annotation | fold change |
| --- | --- | --- | --- | --- |
| Amino acids biosynthesis | *XC_0474* |  | threonine aldolase | 2.01 |
|  | *XC_0839* | *ilvM* | acetolactate synthase isozyme II small subunit | 3.43 |
|  | *XC_0840* | *ilvG* | acetolactate synthase isozyme II large subunit | 2.17 |
|  | *XC_0841* | *ilvC* | ketol-acid reductoisomerase | 2.68 |
|  | *XC_1344* | *nifS* | cysteine desulfurase | 4.15 |
|  | *XC_0982* | *cysK* | cysteine synthase | -2.72 |
|  | *XC_1090* | *metB* | homocysteine synthase | -2.05 |
| Biosynthesis of cofactors, prosthetic groups, carriers | *XC_2553* | *bioC* | biotin synthesis protein | 2.03 |
|  | *XC_3644* | *ptpS* | 6-pyruvoyl tetrahydrobiopterin synthase | 2.93 |
|  | *XC_3541* | *ribD* | riboflavin-specific deaminase/5-amino-6-uracil reductase | -2.22 |
|  | *XC_3751* | *entB* | isochorismatase-like protein | -2.34 |
| Cell envelope and cell structure | *XC_2151* |  | L-sorbosone dehydrogenase | -2.32 |
| Cellular processes | *XC_0638* | *tsr* | chemotaxis protein | -3.71 |
|  | *XC_1409* | *cheB* | protein-glutamate methylesterase | -2.4 |
|  | *XC_1410* | *cheR* | response regulator for chemotaxis | -7.49 |
|  | *XC_1412* | *cheW* | chemotaxis protein | -5.39 |
|  | *XC_1413* | *mcp* | chemotaxis protein | -3.62 |
|  | *XC_1414* | *cheA* | chemotaxis histidine protein kinase | -3.05 |
|  | *XC_1801* | *mcp* | chemotaxis protein | -2.09 |
|  | *XC_2223* | *mcp* | chemotaxis protein | -5.53 |
|  | *XC_2231* | *flgM* | flagellar protein | -7.93 |
|  | *XC_2233* | *cheV* | chemotaxis protein | -3.65 |
|  | *XC_2245* | *fliC* | flagellar protein | -8.82 |
|  | *XC_2246* | *fliD* | flagellar protein | -2.36 |
|  | *XC_2247* | *fliS* | flagellar protein | -6.94 |
|  | *XC_2263* | *fliI* | flagellar protein | -2.27 |
|  | *XC_2264* | *fliJ* | flagellar FliJ protein | -2.86 |
|  | *XC_2265* | *fliK* | flagellar protein | -4.36 |
|  | *XC_2284* | *cheA* | chemotaxis related protein | -2.07 |
|  | *XC_2297* | *motA* | MotA protein | -3.31 |
|  | *XC_2298* | *motB* | MotB protein | -4.63 |
|  | *XC_2299* | *parA* | chromosome partioning protein | -6.25 |
|  | *XC_2300* | *cheW* | chemotaxis protein | -5.55 |
|  | *XC_2302* | *cheY* | chemotaxis response regulator | -5.49 |
|  | *XC_2303* | *cheA* | chemotaxis protein | -5.61 |
|  | *XC_2304* | *tsr* | chemotaxis protein | -5.57 |
|  | *XC_2306* | *tsr* | chemotaxis protein | -13.25 |
|  | *XC_2307* | *tsr* | chemotaxis protein | -6.02 |
|  | *XC_2308* | *tsr* | chemotaxis protein | -2.52 |
|  | *XC_2309* | *tsr* | chemotaxis protein | -10.34 |
|  | *XC_2311* | *tsr* | chemotaxis protein | -15.15 |
|  | *XC_2313* | *tsr* | chemotaxis protein | -4.15 |
|  | *XC_2315* |  | chemotaxis protein | -3.86 |
|  | *XC_2316* | *tsr* | chemotaxis protein | -5.21 |
|  | *XC_2318* | *cheW* | chemotaxis protein | -8.59 |
|  | *XC_2320* | *tsr* | chemotaxis protein | -66.19 |
|  | *XC_2321* | *cheR* | chemotaxis protein methyltransferase | -15.23 |
|  | *XC_2322* | *cheD* | chemotaxis protein | -5.39 |
|  | *XC_2323* | *cheB* | glutamate methylesterase | -7.78 |
|  | *XC_2504* | *mcpA* | chemotaxis protein | -2.61 |
|  | *XC_3724* | *motA* | chemotaxis protein | -4.39 |
|  | *XC_3725* | *motB* | chemotaxis MotB protein | -4.42 |
| Central intermediary metabolism | *XC_1552* |  | carboxymethylenebutenolidase | 10.05 |
|  | *XC_4015* | *argI* | arginase | 2.69 |
|  | *XC_4198* | *mtlD* | mannitol dehydrogenase | 2.40 |
|  | *XC_0150* |  | L-fucose dehydrogenase | -2.35 |
|  | *XC_0308* | *vanA* | vanillate O-demethylase oxygenase subunit | -3.19 |
|  | *XC_0380* | *pcaG* | protocatechuate 3,4-dioxygenase alpha chain | -2.07 |
|  | *XC_0990* | *cysH* | phosphoadenosine phosphosulfate reductase | 2.10 |
|  | *XC_0991* | *cysI* | NADPH-sulfite reductase iron-sulfur protein | -2.64 |
|  | *XC_0993* | *cysD* | ATP sulfurylase small subunit | -3.17 |
|  | *XC_0994* | *cysN* | ATP sulfurylase | -2.94 |
|  | *XC_1642* | *aglA* | alpha-glucosidase | -2.49 |
|  | *XC_1645* | *aglA* | alpha-glucosidase | -2.64 |
|  | *XC_2480* | *xylS* | alpha-xylosidase | -2.30 |
|  | *XC_2482* |  | sialic acid-specific 9-O-acetylesterase | -2.12 |
|  | *XC_2487* | *dgoA* | 4-hydroxy-2-oxoglutarate aldolase | -2.01 |
|  | *XC_2488* | *dgoA* | 4-hydroxy-2-oxoglutarate aldolase | -2.13 |
|  | *XC_3215* | *prpB* | carboxyphosphonoenolpyruvate phosphonomutase | -2.06 |
|  | *XC_3491* | *dgd* | D-galactose 1-dehydrogenase | -5.75 |
| Energy and carbon metabolism | *XC_1698* |  | D-amino acid oxidase | 3.19 |
|  | *XC_1891* | *cycL* | c-type cytochrome biogenesis protein | 2.61 |
|  | *XC_0060* |  | NAD(P)H oxidoreductase | -2.05 |
|  | *XC_0296* |  | oxidoreductase | -2.34 |
|  | *XC_1300* |  | quinol oxidase, subunit I | -2.79 |
|  | *XC_1446* | *mocA* | oxidoreductase | -5.03 |
|  | *XC_3488* |  | phosphoglycerate mutase | -2.42 |
| Fatty acid and phospholipid metabolism | *XC_0384* |  | hydrolase | 2.56 |
|  | *XC_1682* | *blc* | outer membrane lipoprotein | 2.6 |
|  | *XC_1988* | *lolC* | lipoprotein releasing system transmembrane protein | 2.32 |
|  | *XC_2528* | *ispF* | 2C-methyl-D-erythritol 2,4-cyclodiphosphate synthase | 2.14 |
|  | *XC_4301* | *vacJ* | lipoprotein | 2.48 |
|  | *XC_4263* | *cls* | cardiolipin synthetase | -3.30 |
| Nucleotide transport and metabolism | *XC_4075* | *nrdB* | ribonucleoside-diphosphate reductase beta chain | 2.17 |
|  | *XC_0718* |  | bifunctional NMN adenylyltransferase | -2.53 |
| Regulatory functions | *XC_0072* |  | transcriptional regulator | 2.34 |
|  | *XC_0373* | *glpR* | glycerol-3-phosphate regulon repressor | 39.81 |
|  | *XC_1145* | *exsB* | transcriptional regulator | 2.23 |
|  | *XC_1435* |  | transcriptional regulator | 2.37 |
|  | *XC_3375* |  | transcriptional regulator | 2.63 |
|  | *XC_1280* | *soxR* | transcriptional regulator soxR family | -3.58 |
|  | *XC_1489* |  | transcriptional regulator | -2.08 |
|  | *XC_1766* | *rrpX* | transcriptional regulator | -5.84 |
|  | *XC_2157* |  | transcriptional regulator | -3.11 |
|  | *XC_2430* |  | Predicted transcriptional regulator | -2.55 |
|  | *XC_2841* |  | transcriptional regulator | -4.11 |
| Replication and DNA metabolism | *XC_1327* |  | replication related protein | 2.37 |
|  | *XC_1402* |  | pirin-related protein | 2.31 |
|  | *XC_1628* | *uvrB* | excinuclease ABC subunit B | 2.06 |
|  | *XC_2785* |  | helicase | -4.22 |
|  | *XC_3597* |  | DNA-binding protein | -2.14 |
|  | *XC_4203* |  | histone | -2.00 |
| Transport | *XC_0084* | *proP* | proline/betaine transporter | 2.24 |
|  | *XC_0820* | *dctA* | C4-dicarboxylate transport protein | 2.03 |
|  | *XC_1112* | *bfeA* | ferric enterobactin receptor | 2.42 |
|  | *XC_1139* | *tolR* | TolR protein | 2.03 |
|  | *XC_2708* | *pstS* | ABC transporter phosphate binding protein | 2.26 |
|  | *XC_4146* | *ppa* | solute:Na^+^ symporter | 4.12 |
|  | *XC_0911* | *modB* | molybdate transport permease protein | -4.63 |
|  | *XC_1087* |  | ABC transporter ATP-binding subunit | -2.14 |
|  | *XC_1115* | *bfeA* | ferric enterobactin receptor | -2.07 |
|  | *XC_1241* | *btuB* | TonB-dependent receptor | -2.17 |
|  | *XC_1263* | *emrA* | MFS transporter | -2.05 |
|  | *XC_1444* | *araJ* | MFS transporter | -2.08 |
|  | *XC_1644* | *btuB* | TonB-dependent receptor | -3.57 |
|  | *XC_1647* | *suc1* | transport protein | -2.04 |
|  | *XC_2178* | *nasA* | nitrate transporter | -2.5 |
|  | *XC_2484* |  | TonB-dependent receptor | -3.63 |
|  | *XC_3559* |  | putative siderophore receptor | -2.1 |
| Translation | *XC_1292* |  | endoproteinase Arg-C | 2.31 |
|  | *XC_1606* | *rbfA* | ribosomal-binding factor A | 2.61 |
|  | *XC_2929* | *slyD* | peptidyl-prolyl cis-trans isomerase | 3.36 |
|  | *XC_1282* |  | peptidase | -2.03 |
|  | *XC_4009* |  | peptidase | -2.19 |
| *Transcription* |  |  |  |  |
| Signal transduction | *XC_2229* |  | histidine kinase | 2.83 |
|  | *XC_3273* | *phoR* | two-component system sensor protein | 2.15 |
|  | *XC_0637* |  | histidine kinase/response regulator hybrid protein | -6.85 |
|  | *XC_1160* |  | response regulator | -2.13 |
|  | *XC_1421* | *creC* | two-component system sensor protein | -2.49 |
|  | *XC_2275* |  | GGDEF family protein | -3.68 |
|  | *XC_2276* |  | GGDEF family protein | -2.33 |
| Mobile genetic elements | *XC_0412* | *IS1404* | IS1404 transposase ORFA | 9.19 |
|  | *XC_0858* | *IS1477* | IS1477 transposase | 3.21 |
|  | *XC_1034* | *IS1478* | ISxcc1 transposase | 3.47 |
|  | *XC_2111* | *gVIII* | major coat protein | 22.43 |
|  | *XC_2625* | *IS1404* | IS1404 transposase | 71.82 |
|  | *XC_0697* | *IS1404* | IS1404 transposase | -2.04 |
|  | *XC_0909* | *IS1480* | IS1480 transposase | -6.92 |
|  | *XC_1211* | *ISxac3* | ISxac3 transposase | -287.79 |
|  | *XC_2109* | *gVII* | minor coat protein | -44.68 |
|  | *XC_2124* | *gII* | phage-related protein | -2.13 |
|  | *XC_2417* |  | plasmid mobilization protein | -2.26 |
|  | *XC_2434* |  | phage associated protein | -3.91 |
|  | *XC_2780* | *int* | phage-related integrase | -2.58 |
|  | *XC_2784* | *IS1478* | IS1478 transposase | -2.49 |
|  | *XC_3671* | *IS1404* | IS1404 transposase | -78.99 |
| Pathogenicity and adaptation | *XC_0056* |  | hemolysin III | 2.59 |
|  | *XC_0705* | *peh-1* | endopolygalacturonase | 2.04 |
|  | *XC_0755* | *cspA* | nisin-resistance protein | 2.6 |
|  | *XC_1005* |  | 1,4-beta-cellobiosidase | 9.89 |
|  | *XC_1217* |  | glucan 1,4-beta-glucosidase | 2.26 |
|  | *XC_1515* |  | extracellular protease | 3.41 |
|  | *XC_1662* | *gumF* | GumF protein | 2.07 |
|  | *XC_1828* | *cspA* | major cold shock protein | 2.17 |
|  | *XC_1865* | *tetV* | drug:proton antiporter | 2.03 |
|  | *XC_2081* | *avrBs1* | avirulence protein | 2.54 |
|  | *XC_3001* | *hpa2* | Hpa2 protein | 2.21 |
|  | *XC_3017* | *hrcS* | HrcS protein | 59.81 |
|  | *XC_3314* | *dsbB* | disulfide bond formation protein B | 2.77 |
|  | *XC_3377* |  | extracellular protease | 2.44 |
|  | *XC_3378* |  | extracellular protease | 2.23 |
|  | *XC_3590* | *pel* | pectate lyase | 2.16 |
|  | *XC_4073* |  | thioredoxin | 3.52 |
|  | *XC_4126* | *czcB* | cation efflux system protein | 2.1 |
|  | *XC_4127* | *czcC* | cation efflux system protein | 2.23 |
|  | *XC_0456* | *phaF* | PhaF protein | -2.61 |
|  | *XC_0744* | *xcsI* | type II secretion system protein I | -2.24 |
|  | *XC_1411* | *vieA* | response regulator | -2.91 |
|  | *XC_1918* | *pat* | phosphinothricin N-acetyltransferase | -2.02 |
|  | *XC_2324* | *pdeA* | c-di-GMP phosphodiesterase A | -3.64 |
|  | *XC_2483* |  | cellulase | -2.9 |
|  | *XC_3007* | *hrpB5* | HrpB5 protein | -2.13 |
|  | *XC_3098* |  | pathogenicity-related protein | -2.3 |
| Undefined category | *XC_1840* |  | phosphate-binding protein | 2.06 |
|  | *XC_0600* |  | putative transmembrane protein | -4.02 |
|  | *XC_1945* |  | transport protein | -2.13 |
|  | *XC_2433* |  | ATPases of the AAA+ class | -2.36 |
| conserved hypothetical protein | *XC_0105* |  | conserved hypothetical protein | 9.12 |
|  | *XC_0107* |  | conserved hypothetical protein | 2.40 |
|  | *XC_0117* |  | conserved hypothetical protein | 2.04 |
|  | *XC_0335* |  | conserved hypothetical protein | 3.18 |
|  | *XC_0565* |  | conserved hypothetical protein | 2.39 |
|  | *XC_0792* |  | conserved hypothetical protein | 2.30 |
|  | *XC_0854* |  | conserved hypothetical protein | 2.15 |
|  | *XC_0935* |  | conserved hypothetical protein | 2.71 |
|  | *XC_1072* |  | conserved hypothetical protein | 2.45 |
|  | *XC_1073* |  | conserved hypothetical protein | 4.92 |
|  | *XC_1231* |  | conserved hypothetical protein | 2.14 |
|  | *XC_1351* |  | conserved hypothetical protein | 2.56 |
|  | *XC_1438* |  | conserved hypothetical protein | 6.93 |
|  | *XC_1466* |  | conserved hypothetical protein | 2.37 |
|  | *XC_1551* |  | conserved hypothetical protein | 5.66 |
|  | *XC_1782* |  | conserved hypothetical protein | 2.32 |
|  | *XC_1895* |  | conserved hypothetical protein | 112.78 |
|  | *XC_1986* |  | conserved hypothetical protein | 2.47 |
|  | *XC_2141* |  | conserved hypothetical protein | 2.29 |
|  | *XC_2350* |  | conserved hypothetical protein | 2.65 |
|  | *XC_2535* |  | conserved hypothetical protein | 2.07 |
|  | *XC_2631* |  | conserved hypothetical protein | 2.76 |
|  | *XC_2632* |  | conserved hypothetical protein | 3.93 |
|  | *XC_2633* |  | conserved hypothetical protein | 4.46 |
|  | *XC_2634* |  | conserved hypothetical protein | 7.23 |
|  | *XC_2647* |  | conserved hypothetical protein | 2.12 |
|  | *XC_2817* |  | conserved hypothetical protein | 2.03 |
|  | *XC_2872* |  | conserved hypothetical protein | 2.18 |
|  | *XC_2909* |  | conserved hypothetical protein | 5.27 |
|  | *XC_2911* |  | conserved hypothetical protein | 2.52 |
|  | *XC_3533* |  | conserved hypothetical protein | 71.51 |
|  | *XC_3647* |  | conserved hypothetical protein | 2.21 |
|  | *XC_3693* |  | conserved hypothetical protein | 2.58 |
|  | *XC_3826* |  | conserved hypothetical protein | 2.26 |
|  | *XC_3828* |  | conserved hypothetical protein | 2.13 |
|  | *XC_3866* |  | conserved hypothetical protein | 3.90 |
|  | *XC_3869* |  | conserved hypothetical protein | 2.95 |
|  | *XC_3899* |  | conserved hypothetical protein | 3.52 |
|  | *XC_3962* |  | conserved hypothetical protein | 2.28 |
|  | *XC_4050* |  | conserved hypothetical protein | 3.08 |
|  | *XC_4074* |  | conserved hypothetical protein | 2.55 |
|  | *XC_4147* |  | conserved hypothetical protein | 3.22 |
|  | *XC_4148* |  | conserved hypothetical protein | 2.20 |
|  | *XC_4319* |  | conserved hypothetical protein | 3.68 |
|  | *XC_0199* |  | hypothetical protein | 2.58 |
|  | *XC_1390* |  | hypothetical protein | 3.59 |
|  | *XC_1984* |  | hypothetical protein | 10.62 |
|  | *XC_0034* |  | conserved hypothetical protein | -2.22 |
|  | *XC_0053* |  | conserved hypothetical protein | -2.02 |
|  | *XC_0069* |  | conserved hypothetical protein | -2.03 |
|  | *XC_0070* |  | conserved hypothetical protein | -4.11 |
|  | *XC_0071* |  | conserved hypothetical protein | -2.56 |
|  | *XC_0230* |  | conserved hypothetical protein | -44.86 |
|  | *XC_0251* |  | conserved hypothetical protein | -2.23 |
|  | *XC_0258* |  | conserved hypothetical protein | -2.13 |
|  | *XC_0262* |  | conserved hypothetical protein | -33.11 |
|  | *XC_0288* |  | conserved hypothetical protein | -5.89 |
|  | *XC_0362* |  | conserved hypothetical protein | -3.79 |
|  | *XC_0564* |  | conserved hypothetical protein | -3.41 |
|  | *XC_0597* |  | conserved hypothetical protein | -2.45 |
|  | *XC_0613* |  | conserved hypothetical protein | -2.06 |
|  | *XC_0632* |  | conserved hypothetical protein | -2.29 |
|  | *XC_0774* |  | conserved hypothetical protein | -18.57 |
|  | *XC_0780* |  | conserved hypothetical protein | -2.14 |
|  | *XC_0781* |  | conserved hypothetical protein | -2.21 |
|  | *XC_0861* |  | conserved hypothetical protein | -4.19 |
|  | *XC_0870* |  | conserved hypothetical protein | -2.28 |
|  | *XC_0989* |  | conserved hypothetical protein | -2.13 |
|  | *XC_1089* |  | conserved hypothetical protein | -2.17 |
|  | *XC_1148* |  | conserved hypothetical protein | -2.42 |
|  | *XC_1202* |  | conserved hypothetical protein | -12.2 |
|  | *XC_1302* |  | conserved hypothetical protein | -2.16 |
|  | *XC_1323* |  | conserved hypothetical protein | -2.26 |
|  | *XC_1340* |  | conserved hypothetical protein | -2.34 |
|  | *XC_1405* |  | conserved hypothetical protein | -4.30 |
|  | *XC_1415* |  | conserved hypothetical protein | -6.46 |
|  | *XC_1425* |  | conserved hypothetical protein | -4.29 |
|  | *XC_1460* |  | conserved hypothetical protein | -17.43 |
|  | *XC_1709* |  | conserved hypothetical protein | -6.01 |
|  | *XC_1870* |  | conserved hypothetical protein | -2.04 |
|  | *XC_2058* |  | conserved hypothetical protein | -2.05 |
|  | *XC_2066* |  | conserved hypothetical protein | -2.46 |
|  | *XC_2069* |  | conserved hypothetical protein | -2.08 |
|  | *XC_2152* |  | conserved hypothetical protein | -2.36 |
|  | *XC_2166* |  | conserved hypothetical protein | -2.51 |
|  | *XC_2224* |  | conserved hypothetical protein | -2.62 |
|  | *XC_2225* |  | conserved hypothetical protein | -2.14 |
|  | *XC_2226* |  | conserved hypothetical protein | -3.25 |
|  | *XC_2230* |  | conserved hypothetical protein | -5.09 |
|  | *XC_2248* |  | conserved hypothetical protein | -2.15 |
|  | *XC_2249* |  | conserved hypothetical protein | -3.19 |
|  | *XC_2301* |  | conserved hypothetical protein | -11.66 |
|  | *XC_2305* |  | conserved hypothetical protein | -50.24 |
|  | *XC_2312* |  | conserved hypothetical protein | -6.13 |
|  | *XC_2317* |  | conserved hypothetical protein | -6.24 |
|  | *XC_2319* |  | conserved hypothetical protein | -19.46 |
|  | *XC_2411* |  | conserved hypothetical protein | -2.43 |
|  | *XC_2415* |  | conserved hypothetical protein | -3.46 |
|  | *XC_2435* |  | conserved hypothetical protein | -3.71 |
|  | *XC_2459* |  | conserved hypothetical protein | -2.93 |
|  | *XC_2463* |  | conserved hypothetical protein | -9.07 |
|  | *XC_2464* |  | conserved hypothetical protein | -18.65 |
|  | *XC_2481* |  | conserved hypothetical protein | -3.07 |
|  | *XC_2548* |  | conserved hypothetical protein | -2.74 |
|  | *XC_2556* |  | conserved hypothetical protein | -2.03 |
|  | *XC_2587* |  | conserved hypothetical protein | -2.11 |
|  | *XC_2781* |  | conserved hypothetical protein | -2.02 |
|  | *XC_2786* |  | conserved hypothetical protein | -4.43 |
|  | *XC_2787* |  | conserved hypothetical protein | -8.29 |
|  | *XC_2788* |  | conserved hypothetical protein | -5.29 |
|  | *XC_2789* |  | conserved hypothetical protein | -3.68 |
|  | *XC_2814* |  | conserved hypothetical protein | -2.92 |
|  | *XC_2861* |  | conserved hypothetical protein | -2.01 |
|  | *XC_2988* |  | conserved hypothetical protein | -2.64 |
|  | *XC_2999* |  | conserved hypothetical protein | -2.53 |
|  | *XC_3053* |  | conserved hypothetical protein | -2.11 |
|  | *XC_3108* |  | conserved hypothetical protein | -10.47 |
|  | *XC_3110* |  | conserved hypothetical protein | -2.51 |
|  | *XC_3218* |  | conserved hypothetical protein | -49.71 |
|  | *XC_3558* |  | conserved hypothetical protein | -2.09 |
|  | *XC_3577* |  | conserved hypothetical protein | -2.62 |
|  | *XC_3686* |  | conserved hypothetical protein | -2.27 |
|  | *XC_3715* |  | conserved hypothetical protein | -2.04 |
|  | *XC_3723* |  | conserved hypothetical protein | -4.04 |
|  | *XC_3756* |  | conserved hypothetical protein | -2.36 |
|  | *XC_3764* |  | conserved hypothetical protein | -2.36 |
|  | *XC_3765* |  | conserved hypothetical protein | -2.43 |
|  | *XC_3805* |  | conserved hypothetical protein | -2.73 |
|  | *XC_3872* |  | conserved hypothetical protein | -2.62 |
|  | *XC_3956* |  | conserved hypothetical protein | -2.17 |
|  | *XC_3969* |  | conserved hypothetical protein | -3.04 |
|  | *XC_3970* |  | conserved hypothetical protein | -2.73 |
|  | *XC_3976* |  | conserved hypothetical protein | -2.13 |
|  | *XC_3977* |  | conserved hypothetical protein | -2.44 |
|  | *XC_4007* |  | conserved hypothetical protein | -2.02 |
|  | *XC_4035* |  | conserved hypothetical protein | -4.91 |
|  | *XC_4039* |  | conserved hypothetical protein | -3.31 |
|  | *XC_4174* |  | conserved hypothetical protein | -5.40 |
|  | *XC_4242* |  | conserved hypothetical protein | -2.70 |
|  | *XC_1294* |  | hypothetical protein | -3.95 |
|  | *XC_1420* |  | hypothetical protein | -3.69 |
|  | *XC_1458* |  | hypothetical protein | -42.33 |
|  | *XC_1991* |  | hypothetical protein | -9.10 |
|  | *XC_2414* |  | hypothetical protein | -2.15 |
|  | *XC_2416* |  | hypothetical protein | -2.88 |
|  | *XC_2432* |  | hypothetical protein | -2.42 |
|  | *XC_3486* |  | hypothetical protein | -2.41 |
|  | *XC_3950* |  | hypothetical protein | -12.83 |

Note: False discovery rate (FDR) ≤0.05 and absolute value of log_2_ fold change (log_2_ FC) ≥1 (equivalent to a fold change of 2) were used as the cut off values.“+” represents gene up-regulated in the ∆*mcvR* mutant, and “-”represents gene down-regulated.
