# Supplementary material for: McvR, a single domain response regulator regulates motility and virulence in the plant pathogen Xanthomonas campestris
Source: Mol Plant Pathol. 2022 Feb 13;23(5):649–63. doi: 10.1111/mpp.13186 (PMC8995066; doi:10.1111/mpp.13186)
Supplement: Supplementary file 7 — TABLE S3 Confirmation of RNA‐Seq gene expression level by end‐point reverse transcription‐PCR [file MPP-23-649-s001.docx]

**Table S3.** Confirmation of RNA‐Seq gene expression level by end-point RT‐PCR

| ID | Gene | Annotation | Expresstion level | Semi RT-PCR wt/∆*mcvR* |
| --- | --- | --- | --- | --- |
| *XC_2231* | *flgM* | flagellar protein | 7.93↓ | 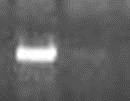 |
| *XC_2245* | *fliC* | flagellar protein | 8.82↓ | 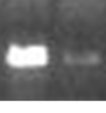 |
| *XC_2247* | *fliS* | flagellar protein | 6.94↓ | 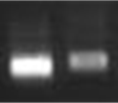 |
| *XC_2302* | *cheY* | chemotaxis response regulator | 5.49↓ | 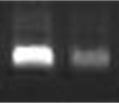 |
| *XC_0993* | *cysD* | ATP sulfurylase small subunit | 3.17↓ | 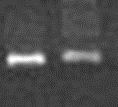 |
| *XC_3597* |  | DNA-binding protein | 2.14↓ | 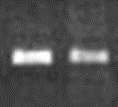 |
| *XC_0820* | *dctA* | C4-dicarboxylate transport protein | 2.03↑ | 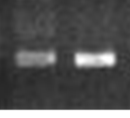 |
| *XC_1087* |  | ABC transporter ATP-binding subunit | 2.14↓ | 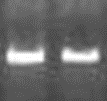 |
| *XC_1292* |  | endoproteinase Arg-C | 2.31↑ | 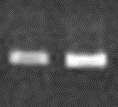 |
| *XC_0705* | *peh-1* | endopolygalacturonase | 2.04↑ | 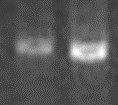 |
| *XC_1005* |  | 1,4-beta-cellobiosidase | 9.89↑ | 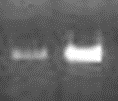 |
| *XC_3001* | *hpa2* | Hpa2 protein | 2.21↑ | 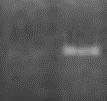 |
| *XC_3377* |  | extracellular protease | 2.44↑ | 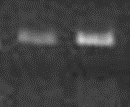 |
| *XC_0744* | *xcsI* | type II secretion system protein I | 2.24↓ | 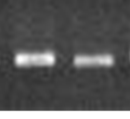 |
| *XC_2324* | *pdeA* | c-di-GMP phosphodiesterase A | 3.64↓ | 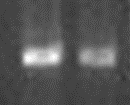 |
| *XC_2631* |  | conserved hypothetical protein | 2.76↑ | 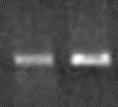 |
| *XC_2634* |  | conserved hypothetical protein | 7.23↑ | 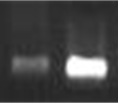 |
| *XC_0251* |  | conserved hypothetical protein | 2.23↓ | 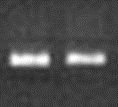 |
| *XC_2166* |  | conserved hypothetical protein | 2.51↓ | 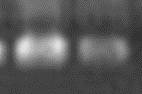 |
| *XC_2416* |  | hypothetical protein | 2.88↓ | 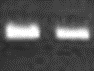 |
| *16S* |  |  |  | 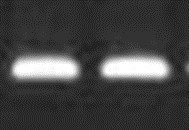 |

Note: RNA samples were processed using the same treatments as for RNA-Seq, and cDNA fragments were obtained by using a cDNA Synthesis kit (Invitrogen, Waltham, MA, USA). The 16S rRNA gene of *Xcc* 8004 was used as the internal control to verify the absence of significant variation at cDNA level in the samples. In this study, FDR≤0.05 and absolute value of log_2_ FC≥1 were used as the cut off values. The acquired results were accordant to the transcriptome data.↑: up-regulated; ↓: down-regulated.
