# Supplementary material for: McvR, a single domain response regulator regulates motility and virulence in the plant pathogen Xanthomonas campestris
Source: Mol Plant Pathol. 2022 Feb 13;23(5):649–63. doi: 10.1111/mpp.13186 (PMC8995066; doi:10.1111/mpp.13186)
Supplement: Supplementary file 8 — TABLE S4 Sequence of the primers used in this study [file MPP-23-649-s004.docx]

**Table S4. Primers used in this study^§^**

| Primer | Nucleotide sequence (5′→3′) | The amplified fragment or the utilization |
| --- | --- | --- |
| L*mcvR-*F  L*mcvR-*R | GGGGGATCCCACTCTTGCTCGCCAAACAACTCA  GGGTCTAGAAACTCCACGCAACGCCGACATTGCT | 456-bp DNA sequence upstream of *mcvR* (*XC_1966*), used for constructing the *mcvR* deletion mutant. |
| R*mcvR-*F  R*mcvR-*R | GGGTCTAGAAGCAGCTGACGCATTGTCAGCG  GGGAAGCTTCGGGATGCAGGCATCGTTGT | 287-bp DNA sequence downstream of *mcvR*, used for constructing the *mcvR* deletion mutant. |
| *mcvR-*OF  *mcvR*-OR | GGGGGATCCTTGCGTGGAGTTCGGGTA  GGGAAGCTTTCAGCTGCTGGCACCGCT | 369-bp DNA fragment of the *mcvR* (*XC_1966*) coding sequence. Cloned into the vector pLAFR3 for complementation or expression vector pET-30a for protein overproduction and pull-down assays. |
| pB*mcvR-*F  pB*mcvR-*R | GTGGATCCGTTGCGTGGAGTTCGGGTACTC  GGAAGCTTTCAGCTGCTGGCACCGCTGCG | 369-bp DNA fragment of the *mcvR* gene coding sequence. Cloned into the vector pBBad22K for overexpression. |
| D55-F  D55-R | GTGCTGGcCTATCGCCTGGGCAACGGCCAGAC  AGGCGATAGgCCAGCACGGCAGTATCCGGCGC | Used for *mcvR* site-directed mutagenesis, replacing Asp55 to Ala55. |
| *fliM-*OF  *fliM*-OR | CGCGGATCC ATGAGCGTCAGTGATCTG  CCCAAGCTT TCATTTGCTGGAGTCCTG | 1011-bp DNA fragment of the *fliM* ORF sequence. Used for overexpression and pull-down assays. |
| *cheY*-OF  *cheY*-OR | CCCGAGCTC ATGCGGATCCTGATCGTG  CCCAAGCTT TCAGGCGGTCGCCGCCAG | 378-bp DNA fragment of the *cheY* (*XC_2282*) ORF sequence. Used for overexpression and pull-down assays. |
| *hupB*-OF  *hupB-*OR | GGGGATCCATGAATAAAACCGAATTG  GCGGAAGCTTTTAGTTTACTGCATCCTT | 270-bp DNA fragment of the *hupB* (*XC_3262*) ORF sequence. Used for overexpression and pull-down assays. |
| L*mcvR-*FlagF  L*mcvR-*FlagR | GTGGATCCGAACTTGGTGCCGAAGGT  CGATATCATGATCTTTATAATCACCGTCATGGTCTTTGTAGTCGCTGCTGGCACCGCTGCG | 655-bp DNA fragment containing 240-bp DNA upstream of the *mcvR*, 369-bp McvR-coding sequence, 43-bp Flag-coding sequence and the 3-bp stop codon. Used for constructing *Xcc* strain producing McvR::3×Flag protein. |
| R*mcvR-*FlagF  R*mcvR-*FlagR | TTATAAAGATCATGATATCGACTACAAAGATGACGACGATAAATGACGCATTGTCAGCGGCGA  GGAAGCTT CTGTGGGCGACGGCAGAG | 574-bp DNA fragment containing 43-bp Flag-coding sequence, 3-bp stop codon and 528-bp downstream of the *mcvR* stop codon. Used for constructing *Xcc* strain producing McvR::3×Flag protein. |
| *fliM*-BTF  *fliM*-BTR | GGGGGATCCATGAGCGTCAGTGATCTGCTTTCC  GGGCTCGAGTCATTTGCTGGAGTCCTGGGAGG | 1011-bp DNA fragment of the *FliM* ORF sequence. Cloned into bait vector pBT for bacterial two-hybrid assays. |
| *mcvR*-TRGF  *mcvR*-TRGR | GGGGAATTCTGCGTGGAGTTCGGGTACT  GGGCTCGAGTCAGCTGCTGGCACCGCT | 369-bp DNA fragment of the *mcvR* ORF sequence. Cloned into target vector pTRG for bacterial two-hybrid assays. |
| *cheY*-TRGF  *cheY*-TRGR | CCGGAATTCTGCGGATCCTGATCGTGGA  CCGCTCGAGTCAGGCGGTCGCCGCCAG | 378-bp DNA fragment of the *cheY* ORF sequence. Cloned into target vector pTRG for bacterial two-hybrid assays. |
| 16SF  16SR | GCCTAACACATGCAAGTCGAACGGC  AATATTCCCCACTGCTGCCTCCCG | 325-bp DNA fragment of the 16S rDNA sequence, used for RT-PCR and qRT-PCR. |
| 2231-F  2231-R | CGAAGGCAATCTACCGACCG  AACGCTTCCTTCACCGCCTG | 219-bp DNA fragment spans nucleotides 15 to 233 bp of the *XC_2231*, used for RT-PCR. |
| 2245-F  2245-R | CCAACAGTTCGAGCATGGCG  ATACCGTCGTTGGCGTTGCG | 157-bp DNA fragment spans nucleotides 59 to 215 bp of the *XC_2245*, used for RT-PCR. |
| 2247-F  2247-R | TTCCAATCGTCAGTACGCCG  TGAGATGGCCGACGATTGC | 203-bp DNA fragment spans nucleotides 9 to 211 bp of the *XC_2247*, used for RT-PCR. |
| 2302-F  2302-R | GAGCGCACGTATCTTGGTGG  CAGCATGGGCGTGAACTTGT | 238-bp DNA fragment spans nucleotides 3 to 240 bp of the *XC_2302*, used for RT-PCR. |
| 0993-F  0993-R | GAGGCCGAGAGCATCCACAT  TTGATGTGCACGCGCAGGTC | 239-bp DNA fragment spans nucleotides 37 to 275 bp of the *XC_0993*, used for RT-PCR. |
| 3597-F  3597-R | CAAGAATGCAAAGAAGCAGC  GCGGTATTTCGGTGCAACCT | 214-bp DNA fragment spans nucleotides from 51 to 264 bp of the *XC_3597*, used for RT-PCR. |
| 0820-F  0820-R | GCCTTCGCCGAAAGCCTCAA  ACCACCATGCCGACGATCAA | 200-bp DNA fragment spans nucleotides 118 to 317 bp of the *XC_0820*, used for RT-PCR. |
| 1087-F  1087-R | CCACAAGGAACTGCATGTCCGTGCG  GATCACCACGATGCGGTCAGCCAGT | 177-bp DNA fragment spans nucleotides 40 to 216 bp of the *XC_1087*, used for RT-PCR. |
| 1292-F  1292-R | GCGTTGTATCTCGCGTTGTTCTCC  AATTGCGCAACACGGGTTGG | 173-bp DNA fragment spans nucleotides 16 to 188 bp of the *XC_1292*, used for RT-PCR. |
| 0705-F  0705-R | AGTTCGCGGCGGCATTGGAA  GGCATCCGCCACTCTTGGAA | 252-bp DNA fragment spans nucleotides 161 to 412 bp of the *XC_0705*, used for RT-PCR. |
| 1005-F  1005-R | CGCTCAAGGCGAAAATGGGTG  TGGCGGTGATGGGTGTGTTG | 165-bp DNA fragment spans nucleotides 5 to 169 bp of the *XC_1005*, used for RT-PCR. |
| 3001-F  3001-R | GACGCGATCCATCGCAACAA  CGCGGGCGTTTCGGAATGAT | 219-bp DNA fragment spans nucleotides 133 to 351 bp of the *XC_3001*, used for RT-PCR. |
| 3377-F  3377-R | AGGTGAATCTGTCCGGGCTG  TCTTGAGCACTTCGGAGCCC | 204-bp DNA fragment spans nucleotides 101 to 304 bp of the *XC_3377*, used for RT-PCR. |
| 0744-F  0744-R | CAGGGTTCTCGCTGCTGGAA  GCAGTGCTTCAATCGCCTGG | 162-bp DNA fragment spans nucleotides 26 to 187 bp of the *XC_0744*, used for RT-PCR. |
| 2324-F  2324-R | TCAGTGACCAGACCGAAGTC  GTGCAGATCAGCGTCATCAC | 274-bp DNA fragment spans nucleotides 194 to 467 bp of the *XC_2324*, used for RT-PCR. |
| 2631-F  2631-R | AACCTAAGACACCGGCCACG  CAGCTTCAACGCCTTCGCTA | 200-bp DNA fragment spans nucleotides 8 to 207 bp of the *XC_2631*, used for RT-PCR. |
| 2634-F  2634-R | GGTGGGAACTTCAGCCGG  AGCCCCCATCACGGTCAG | 286-bp DNA fragment spans nucleotides 3 to 288 bp of the *XC_2634*, used for RT-PCR. |
| 0251-F  0251-R | TTGTGTCAGCTCACTGGCG  CGAAAGCAGTCACGGAAGGT | 155-bp DNA fragment spans nucleotides 1 to 155 bp of the *XC_0251*, used for RT-PCR |
| 2166-F  2166-R | TCCTGATCCAGGTGCTGCTG  GATCCAGGTCATCGGCCATC | 213-bp DNA fragment spans nucleotides 5 to 217 bp of the *XC_2166*, used for RT-PCR. |
| 2416-F  2416-R | GGTCATCAAATGGATGGCGT  CAGGGCAAGCAAGATGAGAA | 160-bp DNA fragment spans nucleotides 33 to 192 bp of the *XC_2416*, used for RT-PCR. |
| 2245-F  2245-R | CCAGACCATCGGCATCAAC  ATGTCTGCCGCAGTATCGC | 219-bp DNA fragment spans nucleotides 462 to 680 bp of the *fliC*, used for qRT-PCR. |
| 2247-F  2247-R | CAATCGTCAGTACGCCGAG  GTTACCAGCGATCTCACCG | 244-bp DNA fragment spans nucleotides 12 to 255 bp of the *fliS*, used for qRT-PCR. |
| 2265-F  2265-R | ATGCTCAACCCGGCCAACAC  TCCTTGGTGGACTGCTTGGC | 209-bp DNA fragment spans nucleotides 118 to 326 bp of the *fliK*, used for qRT-PCR. |
| 2504-F  2504-R | GACTTCCATGCAGGGCGCTATTG  ATTGGCATCGAGCACCGTGC | 237-bp DNA fragment spans nucleotides 262 to 498 bp of the *mcpA*, used for qRT-PCR. |
| 2322-F  2322-R | CCGTGCAAGTCGACGATGTC  ATAACTGCCATAGCGCGCG | 236-bp DNA fragment spans nucleotides 11 to 246 bp of the *cheD*, used for qRT-PCR. |
| 2233-F  2233-R | CGTCTTCAAGGTGCAGGAAG  TTGACGATGCGTTCCACG | 243-bp DNA fragment spans nucleotides 111 to 353 bp of the *cheV*, used for qRT-PCR. |

^§^The underlined sequences indicate the restriction sites for *Bam*HI, *Eco*RI, *Hin*dIII, *Kpn*I, *Pst*I, *Sac*I, *Xba*I and *Xho*I, respectively. The open rectangle (or square) boxes indicate the altered (or inserted) nucleotides. The long square boxes indicate the 43-bp Flag-coding sequence, and highlights in gray show the complementary sequences.
